# Personalizing driver safety interfaces via driver cognitive factors inference

**DOI:** 10.1038/s41598-024-65144-8

**Published:** 2024-08-05

**Authors:** Emily S. Sumner, Jonathan DeCastro, Jean Costa, Deepak E. Gopinath, Everlyne Kimani, Shabnam Hakimi, Allison Morgan, Andrew Best, Hieu Nguyen, Daniel J. Brooks, Bassam ul Haq, Andrew Patrikalakis, Hiroshi Yasuda, Kate Sieck, Avinash Balachandran, Tiffany L. Chen, Guy Rosman

**Affiliations:** 1 Toyota Research Institute, Los Altos, CA USA; 2 Cambridge, MA USA

**Keywords:** Human behaviour, Computer science

## Abstract

Recent advances in AI and intelligent vehicle technology hold the promise of revolutionizing mobility and transportation through advanced driver assistance systems (ADAS). Certain cognitive factors, such as impulsivity and inhibitory control have been shown to relate to risky driving behavior and on-road risk-taking. However, existing systems fail to leverage such factors in assistive driving technologies adequately. Varying the levels of these cognitive factors could influence the effectiveness and acceptance of ADAS interfaces. We demonstrate an approach for personalizing driver interaction via driver safety interfaces that are are triggered based on the inference of the driver’s latent cognitive states from their driving behavior. To accomplish this, we adopt a data-driven approach and train a recurrent neural network to infer impulsivity and inhibitory control from recent driving behavior. The network is trained on a population of human drivers to infer impulsivity and inhibitory control from recent driving behavior. Using data collected from a high-fidelity vehicle motion simulator experiment, we demonstrate the ability to deduce these factors from driver behavior. We then use these inferred factors to determine instantly whether or not to engage a driver safety interface. This approach was evaluated using leave-one-out cross validation using actual human data. Our evaluations reveal that our personalized driver safety interface that captures the cognitive profile of the driver is more effective in influencing driver behavior in yellow light zones by reducing their inclination to run through them.

Improvements in advanced driver safety assistance systems have the potential to save lives^1,2^. However, these safety systems could benefit from targeting *the cause* of individual drivers’ dangerous driving behavior, which is known to be affected by many different factors, including cognitive, social, and situational^3–5^. Among the cognitive factors that influence risky driving behavior are *cognitive impulsivity*, which is the tendency to act without thinking^6^, and *inhibitory control*, which is the ability to suppress goal-irrelevant stimuli and behavioral responses^7^. Risky driving has been associated with higher self-reported impulsivity^4,8–11^, and with poorer inhibitory control in relevant laboratory tasks^4,10–13^. A recent review has shown the relationship between impulsivity and speeding and other driving violations^14^. More recent work has emphasized that the relationship between impulsive processes and driving errors and violations is influenced by cognitive abilities and self-regulation^15,16^. Further, such effects are associated with both sensation seeking (a concept related to impulsivity) and age, with recent work demonstrating that higher sensation-seeking and younger age were predictive of the highest speed during driving on a virtual reality track^17^. These cognitive factors also influence individuals’ reactions to different types of interfaces^18,19^.

Paaver et al.^20^ showed that even a brief classroom-style lesson on impulsivity and driving can prevent speeding. Although the significance of impulsivity and inhibitory control as risk factors for vehicle accidents has not yet been leveraged in ADAS interfaces, these concepts have been used to develop effective driver educational materials. While there are numerous driver safety interfaces available, there is a gap in the research regarding the influence of impulsivity and inhibitory control on drivers’ responses to these interfaces. More specifically, studies have not adequately explored how to tailor the deployment of these safety interfaces to individual drivers, taking into account their unique levels of impulsivity and inhibitory control. Such personalization is crucial, as it can determine the effectiveness of the interface in enhancing driver safety.

Thus, the efficacy of driver safety systems may vary due to individual differences in cognition. The design of human-machine interfaces (HMIs), with a focus on addressing specific cognitive characteristics, has the potential to enhance both their safety effectiveness and user acceptance^21^. Crucially, the ability to estimate cognitive characteristics from observed driver behavior lays the groundwork for more personalized and effective safety interventions.

Our goal is to build a driver safety system that leverages learned representations of individual drivers’ cognitive factors to personalize HMIs that result in safer driving outcomes. Such a system would allow us to fully separate the underlying reasons for personalization (i.e., the learned cognitive factors) from what specific HMI attributes are personalized as a result of those reasons. This approach, in turn, allows for the deployment of highly versatile safety systems - for instance, if a new HMI is developed, these can be integrated without additional re-training of the underlying representation. The neural representations of cognitive factors enable refinement of the estimated factors, as well as deployment of personalized safety intervention, at large scale.

In this paper, we present experimental evidence for how factors such as impulsivity and inhibitory control can influence people’s responses to driver safety interfaces and how the inference of such cognitive measures enables an approach for personalizing safety interfaces. We do so by constructing a neural network model that embeds driver behavior into a latent space that captures these factors; finally, we demonstrate the embedded representation’s utility for triggering the deployment of assistive driving interfaces targeted to inhibitory control and impulsivity. To our knowledge, we are the first to demonstrate driver assistance personalization in a high-fidelity simulator.

In this paper we contribute: (1) Experimental evidence of how impulsivity and inhibitory control relate to performance under different choices of driver safety systems on a new dataset collected in a large-scale, high-fidelity, driving simulator; (2) A neural network model capable of encoding individual cognitive factor differences based on recent driving behavior; and (3) A decision-making system capable of personalizing the activation of driver safety interface based on the inferred cognitive factors.

## Related works

Our work is at the intersection of two active research areas: the role of cognitive factors in understanding driving behavior, and learning approaches that capture specific latent factors for HMIs.

**Cognitive factors and driving behaviors** Common approaches for assessing driving behavior commonly involve self-report surveys^22^, ticketed speeding violations^23^, or crash records^24^. While these measurements can be good indicators of risky driving behavior, self-report metrics such as these are not always reliable^25^, contain private information, and do not lend themselves to seamless integration into preventative use with drivers. Other studies have shown driving characteristics can be estimated by measuring reactions to predetermined unsafe events in a simulated driving task^12^.

**Figure 1:**
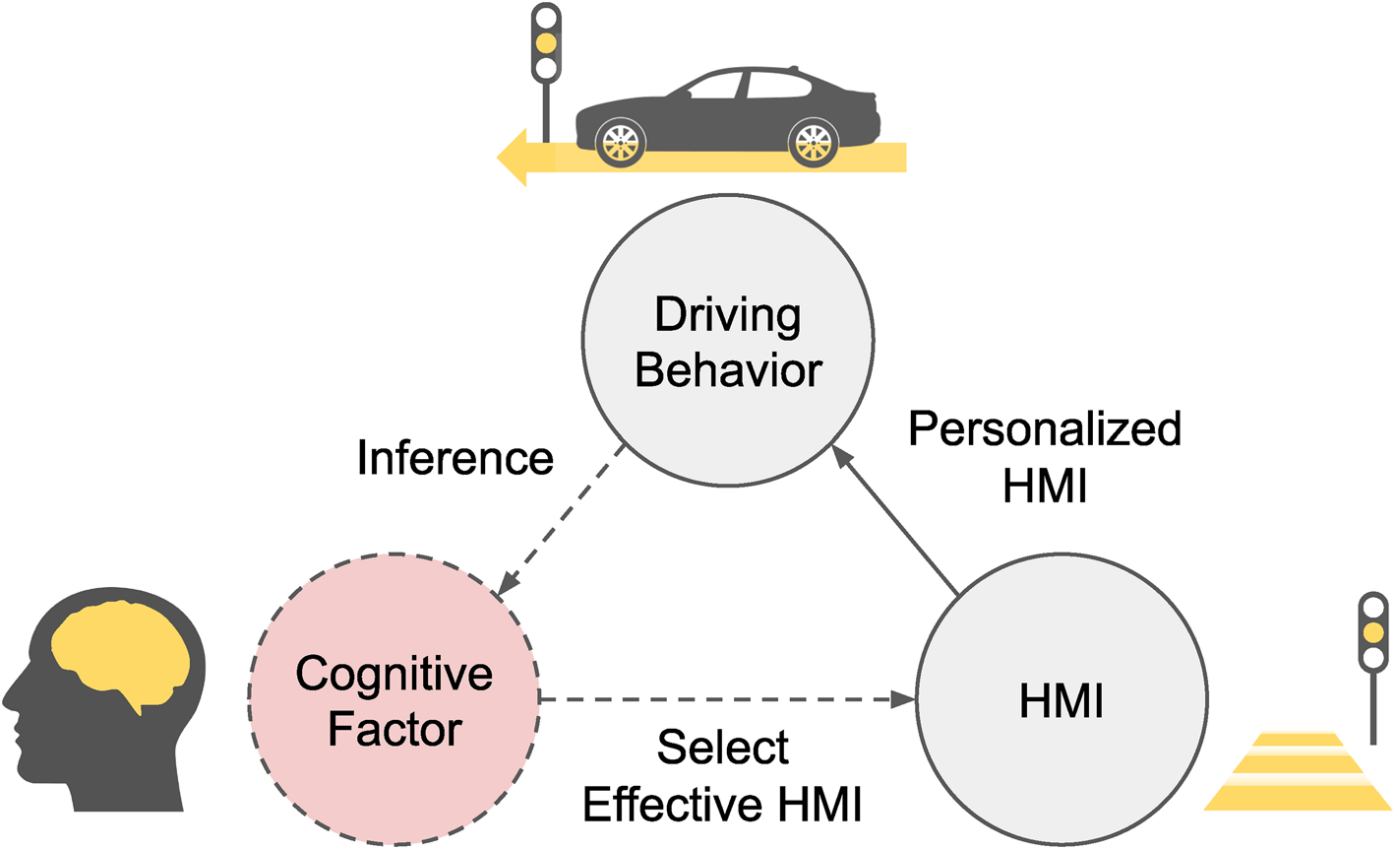
A conceptual overview of our framework. Latent factors embed cognitive measures from the driving behavior, and used to inform HMI choice(dashed lines). Solid line marked the observable driving behavior and personalized HMI.

Our work provides a comprehensive general approach (Fig. 1) to inferring latent cognitive factors from driving behavior logs via a neural network encoder, and uses a high-fidelity driving motion simulator where behavior is closer to real-world vehicles than in lower-fidelity simulators (e.g., bench set up with a steering wheel) (Fig. 3c).

In addition to measuring driving behavior, researchers often measure impulsivity and other behavioral and cognitive factors via tests and questionnaires^26–29^. However, for these cognitive factors to effectively enhance vehicle safety systems, they should be estimated in a scalable way and applied to the development of personalized assistive interfaces within vehicles. In our work, we adopt a data-driven approach to train a neural network model that estimates cognitive factors from *driving behavior* (as opposed to relying on tests and questionnaires) thereby lending itself to deployment at scale. This could lead to more accurate information about drivers and further lead to effective intervention design and deployment criteria.

**Learning Latent Factors for Human-Machine Interfaces** Since an intelligent vehicle is a robotic system, our approach also relates to efforts in personalizing interactions between humans and robots or other machines. Prior work in machine learning for HMIs and human-robot teaming has focused on various human-robot interaction modalities such as driver monitoring, optimal shared control laws, and design of assistive robot behaviors (see e.g.^30–33^). However, these approaches for human-robot interactions typically do not explicitly consider individual differences in cognitive factors and therefore fall under the category of a “one-size-fits-all” design.

The same is true for modern-day driver assistance systems such as lane-departure warnings or forward-collision warnings. Typical interventions issued by such systems depend on an individual’s state and action history and manifest as corrections of unsafe or suboptimal human actions generated from a policy learned from a desired set of behaviors required of the system^34^. Such approaches have been found to over-fit to the average-case behavior of individuals in a population, leading to incorrect inference of the human’s state and poor generalizability^35,36^. Given both the safety risks and the high degree of individual variation in factors like impulsivity and inhibitory control, over-fitting can have potentially dire consequences for drivers^37^. Recent work has shown that learning latent representations summarizing human behavior can improve teaming and interaction with the human. For instance, work on dialog systems^38^, recommender systems^39,40^, and intent recognition for products and motion^41,42^ have demonstrated that latent representations are capable of better predicting the user’s need for a given intervention and their reaction to that intervention. We posit that using this representation as a basis for deciding whether to interact and which modes of interaction to use should improve safety over “one-size-fits-all” decision schemes.

In this paper, we explore how to effectively personalize HMIs based on people’s impulsivity and inhibitory control. We posit that latent factors such as impulsivity and inhibitory control can be inferred in an automated manner from driving behavior and can inform choices of interactions with the drivers to benefit them at a large scale.

## Computational model

**Figure 2:**
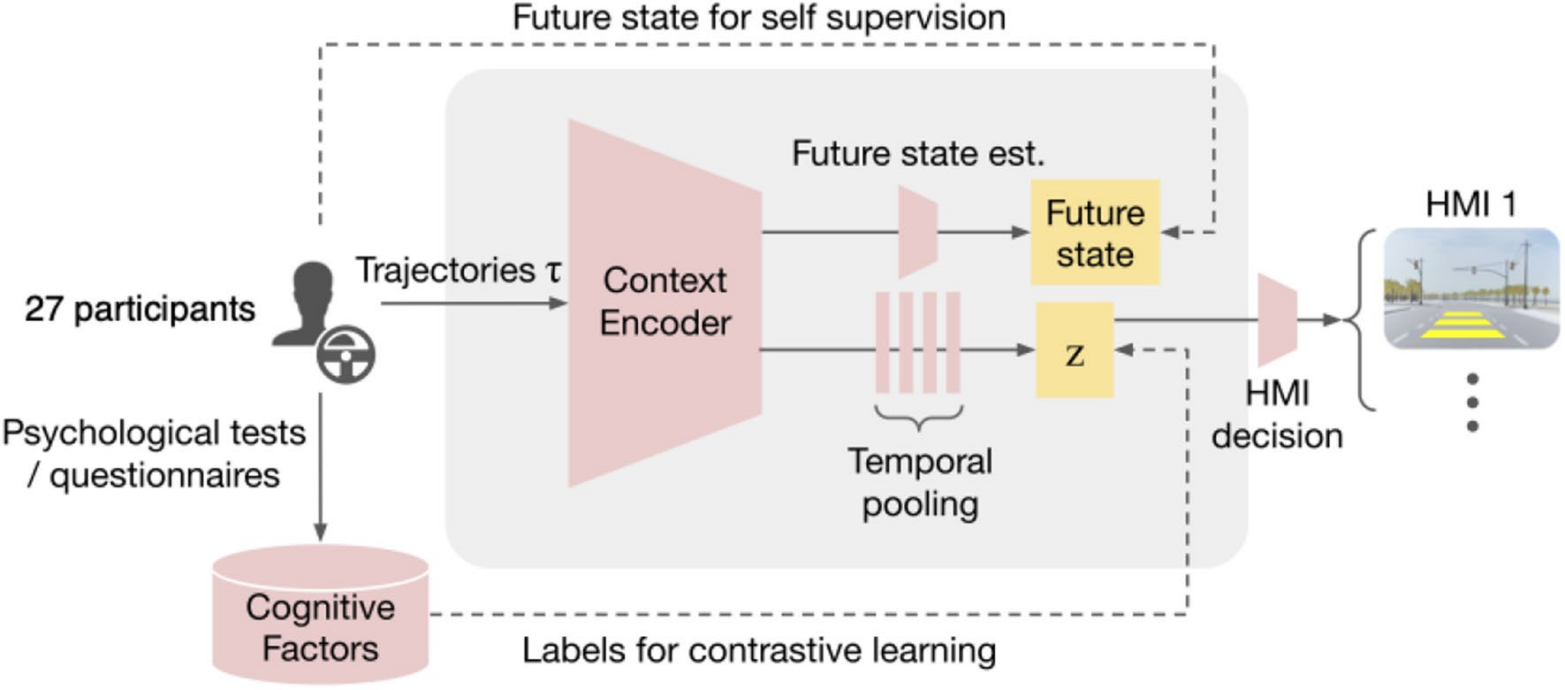
Overall system architecture, including context encoder, decoder for future state and action prediction, outputs of cognitive measures, and latent factors used for HMI selection and decision-making.

We now proceed to describe our computational approach for encoding latent cognitive factors. The resulting neural network distills a human driver’s recent driving history down to a low-dimensional parameter space whose structure can be easily shaped via multiple cognitive measures in a semi-supervised manner. The model we use includes a context encoder whose input is a time-receding, fixed-window trajectory of driving behavior in a scenario and whose output is a low-dimensional latent vector. This latent representation is then coupled with a separate decision-making module that takes this latent vector as input and outputs a decision at each decision time-step; for instance, whether or not to present a particular HMI to the driver at the current time-step. The architecture is shown in Fig. 2, with further details in the “supplemental information”. As a result of experimentation, we found that a two-dimensional latent vector provided sufficient capacity to capture relevant cognitive factors, yet allow direct interpretation of the learned trends in the representation without possible distortions introduced by dimensionality reduction schemes (e.g. t-distributed Stochastic Neighbor Embedding^43^).

The context encoder is represented as a long short-term memory (LSTM) recurrent neural network^44^, $q_{\psi }(z \mid \tau )$, and defines the probability of latent vector *z* given a past trajectory $\tau$ of the driver.

The hidden layer *h* is fed into two linear layers that output the mean and log-variance of the latent encoding^45^.

As driving actions do not directly relate to psychological traits, we leverage *contrastive learning* ^46,47^ to encourage the latent representation to conform to measured cognitive factors (we introduce the specific factors we use in the Results section). As decisions should be based on more than one cognitive factor, we consider our *cognitive factor target* to be a vector.

The context encoding model transforms a driver’s past driving history $\tau$ to a latent vector *z*, and uses a decoder network $p_{\theta }(a|z)$ to predict the driver’s action *a* at the current time-step. We set up the loss terms to encourage *z* to capture both the individual’s cognitive factors and reconstruction of driver actions, with the factors allowing for the downstream decision-making module to have awareness of any time-independent factors inherent to the individual driver, as well as driver actions allowing for awareness of the behaviors in a given situation. Any scene context information present in $\tau$ will indirectly manifests in *z* through $q_{\psi }(z \mid \tau )$. Thus, we expect a weak dependence of predicted driver action on scene context. The overall loss used to train the encoder consists of three components:

- $L_1(a, z; \theta ) = -\mathbb {E}_z \log p_{\theta }(a \vert z)$ is the expected negative log likelihood of action *a* under the model (reconstruction loss) induced by the conditional distribution *p* over *z*, where *z* characterizes driving behavior up to time *t* and $\theta$ represents the parameters of the action decoder network.
- $L_2(z,y;\psi)$, a contrastive loss supervised using a vector of cognitive factor targets *y*^48^. For continuous-valued cognitive measures, this loss is
  $$\begin{aligned} L_2(z,y; \psi ) =&\sum _{z' \in \mathcal {Z}} (1 - \Vert y_{z} - y_{z'} \Vert ^2) \ell (z, z')^2 + \Vert y_{z} - y_{z'} \Vert ^2 \max (0, \epsilon - \ell (z, z'))^2, \end{aligned}$$
  where $\mathcal {Z}$ represents a training samples batch, where each independently-sampled $z, z' \in \mathcal {Z}$ is a $\vert Z \vert$-dimensional latent vector induced by the LSTM context encoder with parameters $\psi$, $y_{z}$ is a vector of batch-normalized cognitive measures associated with *z*, $\ell (z, z')$ is a measure associated with two vectors *z* and $z'$ (which we choose as their Euclidean distance, i.e. $\Vert z - z'\Vert$), and $\epsilon$ controls the magnitude of dissimilarity of *y*-values in *z*-space, where a larger $\epsilon$ enforces higher separation of $\Vert z - z'\Vert$ for fixed $\Vert y_{z} - y_{z'}\Vert$.
- $L_3(z) = D_{KL}(q_{\psi }(z \mid \tau )\vert \mathcal {N}(0,I))$, a Kullback-Leibler (KL)-regularization loss for the distribution of *z*, e.g. as in^49,50^. $\mathcal {N}(0, I)$ is the unit-normal distribution of appropriate dimension.

These terms are combined into an overall training loss:

1 $$\begin{aligned} L(a, z, y; \theta, \psi ) = \alpha _1 L_1(a, z; \theta ) + \alpha _2 L_2(z, y; \psi )+ \alpha _3 L_3(z) \end{aligned}$$

where $\alpha _1$, $\alpha _2$, and $\alpha _3$ are the respective loss coefficients.

**HMI Decision-Making**: We evaluate the utility of the inferred latent factors model by marrying it with a decision rule for selecting the activation of the HMI. The decisions are defined via a simple classifier whose inputs are the inferred latent factors. The classifier is trained to optimize a criterion for HMI selection within the training data. We take the criterion for classification to be the difference in average speed between two conditions, with and without HMI, when the yellow light is active, (averaged across trajectories for a single subject). This criterion reflects the speed reduction induced in the subject when an HMI is presented to the driver. Therefore, for each subject, we have a single regression target and the decision maker is trained to map the latent factors inferred from that subject’s trajectory snippets around yellow light transitions to the corresponding regression target; essentially learning a many-to-one function. We use Support Vector Regression^51^ with a polynomial kernel as our decision model.

## Behavioral experiment

Our motion-simulator driving experiment was designed to address the following hypotheses:

### H1

People with different levels of cognitive factors should exhibit different driving behaviors.

### H2

People with different levels of cognitive factors should respond differently to HMIs.

### H3

Our model should infer individual differences in cognitive factors from driving behavior data.

### H4

When using our model of inferred cognitive factor differences to choose HMIs, and those choices should result in lower speeds when passing through traffic lights.

**Figure 3:**
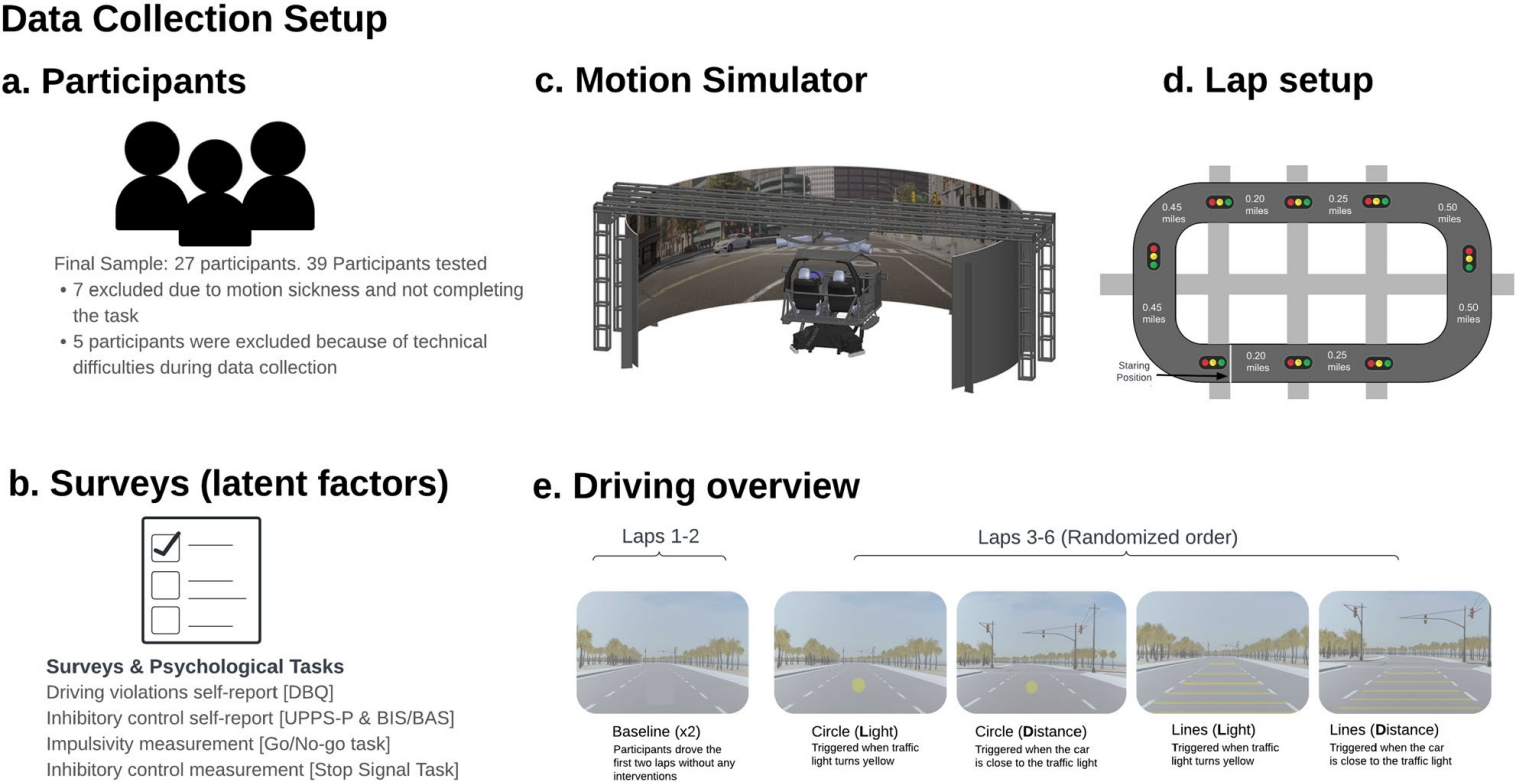
(**a**) Participant overview. (**b**) Set of surveys used to measure latent cognitive factors. (**c**) An illustration of the driving motion simulator used for data collection. (**d**) Driving task course overview. For each lap, four of the lights would transition from green to yellow to red; these were randomly selected for each trial. (**e**). Set of HMIs presented in the driving task. Participants would complete two baseline laps to start. The first baseline lap was considered practice to get the driver acclimated to the simulator and was not included in analysis. After the second baseline lap, the four HMI trials were randomized in the order they were presented to the driver.

The goal of our experiments is to validate H1–H4 by performing the following: (1) constructing candidate HMIs using a simple hand-crafted decision rule to time the deployment of the HMI for alerting the driver when they were approaching a traffic light to influence their driving behavior (specifics can be found in Fig. 3e, (2) data collection of unassisted, baseline driving behaviors from a variety of types of individual drivers in a simulated road setting involving traffic lights, (3) data collection of driving behaviors with the HMI assistance schemes, (4) utilizing the collected data for training a model that encodes cognitive traits, as measured by cognitive assessments, from driving behavior.

In post-hoc, retrospective, analysis of the data, we conducted: (5) post-hoc evaluation of the HMI effect on driver behavior on approach to traffic lights, (6) post-hoc evaluation of our encoding of cognitive traits with respect to cognitive assessments, and (7) post-hoc evaluation of individuals’ behavioral response with the provided HMIs and using the models. Due to the logistical constraints associated with including more participants in our study, we designed our experiments to use a single pool of subjects to address each tasks (1)–(7). Hence, we conduct a randomized study involving each candidate HMIs without using the cognitive inference model. Data collected from the study was used to train a neural network-based cognitive inference model. The model was validated using a leave-one-out cross-validation scheme with respect to a chosen behavior statistic (mean speed during yellow light phase), in retrospect, by averaging over trials in which the experimental condition matched the model’s decision.

### Participants

Thirty-nine Northern California-based drivers aged 18 and older (*Mean age = 49, Female = 16, Non-binary = 1*) were recruited to participate in our study via Fieldwork, a global market research firm. Participants were only invited to participate if they held an active driver’s license, were not pregnant, and were vaccinated for COVID-19. Further details can be found in the recruitment section in the “supplemental information”.

Half of the participants were between the ages of 18–22, the other half were over the age of 65. We chose to recruit these two age groups because previous research has shown significant differences in their levels of impulsivity, inhibitory control, and risk propensity. ^52^ Additionally, these two populations are at heightened risk of vehicle accidents^11^. We opted to start with these groups to determine if there is a detectable signal. While age-related differences are not discussed in this paper, additional analyses can be found in the “supplemental information”. We did not find any significant differences between these two populations in our analysis.

This research was reviewed, approved, and done according to the human-subject guidelines set by the Western Institutional Review Board-Copernicus Group (WCG) IRB protocol number 20221727. Participants filled out a consent form prior to participation and were compensated $150 for their two-hour participation.

#### Exclusion criteria

Participants were excluded from the analysis if they did not complete the study. Of the 39 participants, 7 participants did not complete the driving trials due to motion sickness. Of the 32 remaining participants, the data of 5 participants was excluded from the analysis due to technical difficulties with the motion simulator during testing. The final sample size was therefore 27 individuals.

### Materials

#### Driving task

As illustrated in Fig. 3d, participants drove on a looped road with traffic lights that randomly changed from green to yellow at varying times of arrival of the vehicle at the traffic light, inducing a zone of dilemma^53^ (See Fig.  3d). Each loop consisted of eight traffic lights, four of which would turn yellow. The driving time during the laps summed over all participants was 540 min, which has been shown to be sufficient for driver behavior estimation in similar driving conditions^54^. We collected four driving trials (laps) where participants interacted with different prototype driver safety interfaces and two baseline driving laps without the interfaces.

#### Motion simulator

Participants completed the driving portion of the task using our vehicle motion simulator (See Fig. 3c^55–57^). The motion simulator has a cabin with two car seats, a steering wheel, and pedals that resemble the front half of a vehicle. The cabin is supported by a 6 DOF Motion Platform^58^ and actuated based on the simulated vehicle movement in a virtual traffic environment. The cabin is surrounded by a projection screen that shows the virtual traffic environment. The CARLA simulator controls the virtual traffic and renders high-fidelity visuals by Unreal Engine^59^. A control booth behind the cabin allows the experimenter to control the scenarios and monitor participant safety. Communication between the experimenter and participant is enabled through a headset that is connected to a microphone and speakers in the cabin.

### Method

#### Driver safety interfaces

Two types of warning interfaces were used: a) transverse markings, projected on the road the car was driving; and b) a 2D yellow circle, projected as if it appeared in a heads-up display. Figure 3e shows the virtual scenario and both interface types. The first two laps had no interfaces. The purpose of the first baseline lap was for the participant to get acclimated to the simulator and get a feel for how it drives and not included in analysis. For each interface, we also manipulated a trigger condition that determined whether or not it was displayed. Each interface was displayed either when the vehicle approached the traffic light (185 meters away) or when the upcoming traffic light changed from green to yellow.

- **Impulsivity: ** To assess participants’ impulsivity^60^, we used the **BIS/BAS** scale and the **UPPS-P** scale. The BIS/BAS was used to measure both the behavioral inhibition system (BIS) and the behavioral activation system (BAS), while the UPPS-P was used to account for different facets of impulsivity^61^.
- **Inhibitory Control:** We used the **Go-No Go task**^62^ and the **Stop Signal task **^63,64^ to measure response inhibition. Stop Signal task measures were as described by Verbruggen et al.^64^.
- **Self-reported Driving Behavior: ** To assess participants’ road errors and violations, we used the Manchester Driver Behavior Questionnaire (DBQ)^22^. It includes four sub-scales that measure driver errors (such as failing to check your mirrors), lapses (such as turning the wrong blinker on), aggressive violations (such as racing other vehicles on the street), and ordinary violations (such as ignoring the speed limit on the highway).
- **Driving Behavior in the Motion Simulator: ** We also captured driving behavior as participants drove in the motion simulator. We recorded their driving speed, acceleration, and response to yellow traffic lights.

**Figure 4:**
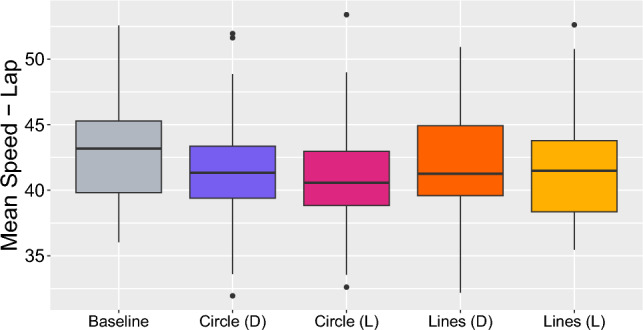
The effect of different HMI types on the mean speed during the lap. “D” refers to a distance-based trigger of the HMI, where the HMI is presented when the vehicle enters within 185 meters of the traffic light, and “L” refers to a light-based trigger, where the HMI is presented at the moment the traffic light turns from green to yellow. Each box plot displays the median, interquartile range (IQR), and outliers for the mean speed during these conditions.

## Results

We analyzed the relationship between various aspects of impulsivity, inhibitory control, driving behavior, and responses to HMIs designed to encourage drivers to slow down. We then analyzed the performance of our model in inferring participants’ cognitive factors and predicting whether they should interact with a HMI to support driving goals.

### Relationship between cognitive factors and driving behavior (H1)

To understand the relationship between the different cognitive factors and driving behavior when reacting to the yellow lights, we conducted a Bayesian correlation analysis using the JASP software^65^. For the analysis, we used the data from all of the driving laps – including the ones with HMIs presented. A table with all of the Bayesian correlations can be found in the “supplemental information” document. As shown in these tables, a number of significant correlations emerged.

The self-reported ordinary violations (errors such as speeding or staying close to another vehicle you are behind) measured in the DBQ^22^ were (mean = 12.778, sd = 1.819) positively correlated with the mean speed at the yellow light (r = 0.4, BF10 = 9693) and the maximum speed when the yellow was active light (r = 0.54 BF10 = $1.141\times 10^9$), indicating that drivers who reported higher levels of ordinary violations from the DBQ (mean = 13.556, sd = 4.348) were more likely to speed through yellow lights in this task.

We found several correlations between the BIS/BAS measures and driving behavior. In particular, BAS Fun Seeking mean = 11.704, sd = 2.165 was positively correlated with the mean speed at the active yellow light (r = 0.473, BF10 = $1.700\times 10^6$) and the maximum speed at the yellow light (r = 0.31, BF10 = 99.19). These data suggest that individuals who have a higher desire for new and exciting experiences may be more likely to take risks while driving, such as speeding through yellow lights. BAS Reward Responsiveness (mean = 16.741, sd = 1.740) was also positively correlated with the maximum speed at an active yellow light (r = 0.29, BF10 = 39.63).

Similar to the BIS/BAS measures, various correlations emerged using the UPPS-P subscales. For instance, UPPS-P Positive Urgency (mean = 6.630, was positively correlated with the maximum speed at an active yellow light (r = 0.28, BF10 = 26.93), and UPPS-P Sensation Seeking (mean = 11.000, sd = 3.150) was positively correlated with the mean speed at the active yellow light (r = 0.29, BF10 = 42.89) and the maximum speed at the active yellow light (r = 0.47, BF10 = $1.540\times 10^6$). These results are consistent with the results found for BAS Fun Seeking (mean = 11.704, sd = 2.165) and BAS Reward Responsiveness (mean = 16.741. sd = 1.740), which provides further evidence that people who desire fun, new and thrilling experiences are more likely to speed and take risks when reacting to traffic lights.

Multiple correlations also emerged using the measures from the Stop Signal task. For instance, the reaction time on go trials with a response (goRT_all, mean = 618.148, sd = 170.594) was negatively correlated with the mean speed at the yellow light (r = $-$ 0.38, BF10 = 2933). This suggests that drivers with longer reaction times may be more likely to slow down at yellow lights rather than speeding through them.

Finally, we also found numerous correlations using the Go/No-Go measures. Among the correlations, the average response time (gonogo_average_rt, mean = 382.981, sd = 49.262) was negatively correlated with the mean speed at the yellow light (r = $-$ 0.46, BF10 = 352747) and the maximum speed at the yellow light (r = $-$ 0.40, BF10 = 9205), which is consistent with the reaction time results from the Stop Signal task (e.g. goRT_all).

**Figure 5:**
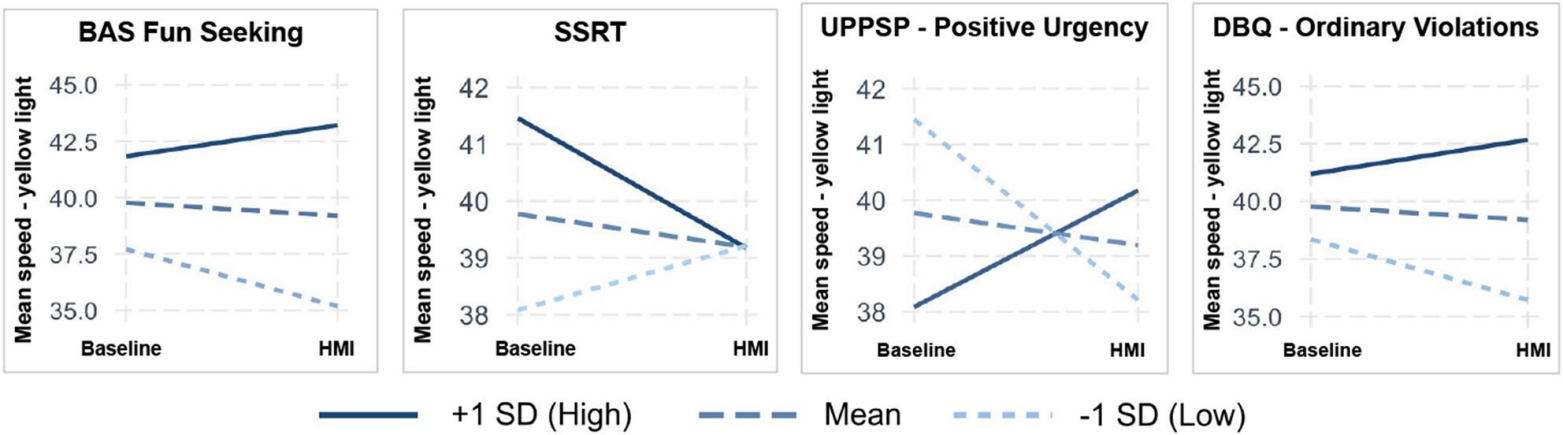
Interaction plots showing how the presence of the HMI interacted with different measures. The lines represent different levels of the measures: +1 SD (High), Mean, and -1 SD (Low). From left to right, the measures are: (**a**) BAS Fun Seeking: Motivation to find novel rewards spontaneously; (**b**) SSRT: Stop Signal Reaction Time: Ability to inhibit a response; (**c**) UPPS-P Positive Urgency: Tendency to act impulsively due to positive affect; d) DBQ Ordinary Violations: Self-reported ordinary driving violations.

### Impact of cognitive factors on people’s driving responses to the interfaces (H2)

We fitted separate linear mixed models to predict each driving behavior measure based on interface condition (Table 1). All conditions demonstrated a statistically significant and negative effect on the mean speed during the lap, as depicted in Fig. 4.

To further understand how different factors affect drivers’ responses to HMI, we conducted a linear mixed models (LMM) analysis, using multiple LMMs to examine the effects of various factors, including the presence or absence of HMI (*HMI_presence*) and their potential interactions. Participant ID was used as a random effect to account for individual differences. The *lmer* function in the *lme4* R package^66^ was employed for predicting mean speed when yellow lights were active based on these variables as

$$\begin{aligned} \text {Mean}\_\text {speed}\_\text {yellow} \sim \text {HMI}\_\text {presence}*\text {Cognitive}\_\text {Factor} + (1 | \text {Participant}), \end{aligned}$$

where $(1 | \text {Participant})$ denotes the random intercept. The models were fitted using the Restricted Maximum Likelihood (REML) estimation method, and the t-tests utilized Satterthwaite’s approximation method.

For detailed statistical outcomes, please refer to Table 1. For a visual representation of some interaction effects, please see Fig. 5, which complements the textual analysis. Here, we highlight some key findings that were noted to have a strong effect:

**BIS/BAS**: The BAS Fun Seeking subscale showed a significant main effect of HMI presence ($\beta = -11.14$, $SE = 4.64$, $t = -2.4$, $p = 0.018$) and a significant interaction with BAS Fun Seeking ($\beta = 0.9$, $SE = 0.39$, $t = 2.31$, $p = 0.023$), suggesting that individuals with higher BAS Fun Seeking scores drove faster in the presence of HMI compared to those with lower scores. The fixed effects accounted for 22.5% of the variance ($R^2_m = 0.225$), while the combined fixed and random effects accounted for 75% ($R^2_c = 0.75$).

**UPPS-P**: The Positive Urgency subscale revealed a significant main effect of HMI presence ($\beta = -8.71$, $SE = 2.66$, $t = -3.28$, $p = 0.0014$) and a significant interaction with Positive Urgency ($\beta = 1.23$, $SE = 0.38$, $t = 3.22$, $p = 0.0017$), indicating that individuals with higher Positive Urgency scores drove faster in the presence of HMI. The fixed effects explained 2.2% of the variance ($R^2_m = 0.022$), while the combined fixed and random effects explained 76.4% ($R^2_c = 0.764$).

**Go/No-Go Measures**: The Go/No-Go Average Response Time measure showed no significant main effect of HMI presence ($\beta = -0.85$, $SE = 6.88$, $t = -0.124$, $p = 0.9019$), but a significant effect of response time ($\beta = -0.072$, $SE = 0.028$, $t = -2.57$, $p = 0.0139$), indicating that longer response times were associated with slower driving speeds. The interaction between HMI presence and response time was not significant ($\beta = 0.00017$, $SE = 0.018$, $t = 0.010$, $p = 0.9922$). The fixed effects explained 20.4% of the variance ($R^2_m = 0.204$), while the combined fixed and random effects explained 74.0% ($R^2_c = 0.740$).

**Stop Signal Measures**: The SSRT measure showed no significant main effects of HMI presence ($\beta = 3.69$, $SE = 2.29$, $t = 1.61$, $p = 0.1094$) or SSRT ($\beta = 0.0145$, $SE = 0.0131$, $t = 1.11$, $p = 0.2724$). However, a significant interaction between HMI presence and SSRT was observed ($\beta = -0.0147$, $SE = 0.0073$, $t = -2.01$, $p = 0.0471$), suggesting that individuals with higher SSRTs drove slower in the presence of HMI compared to those with lower SSRTs. The fixed effects explained 1.0% of the variance ($R^2_m = 0.010$), while the combined fixed and random effects explained 75.1% ($R^2_c = 0.751$).

**Manchester DBQ**: The DBQ Ordinary Violations subscale showed a significant main effect of HMI presence ($\beta = -6.99$, $SE = 2.76$, $t = -2.53$, $p = 0.0128$) and a significant interaction with Ordinary Violations from the DBQ ($\beta = 0.473$, $SE = 0.194$, $t = 2.44$, $p = 0.0164$), suggesting that individuals with higher Ordinary Violations on the DBQ scores drove faster in the presence of HMI. The fixed effects explained 16.4% of the variance ($R^2_m = 0.164$), while the combined fixed and random effects explained 75.2% ($R^2_c = 0.752$).

**Table 1:** Summary of Interaction Effects. This table details the interaction effects between HMI presence and various measures on the cognitive factors.

Interaction Effects	${\beta }$	SE	df	t	p
BAS Reward * HMI_presence	1.48	0.47	106.0	3.095	0.002
BAS Fun Seeking * HMI_presence	0.9	0.39	106.0	2.31	0.02
UPPS-P Positive Urgency * HMI_presence	1.23	0.38	106.0	3.22	0.002
UPPS-P Sensation Seeking * HMI_presence	0.64	0.26	106.0	2.38	0.01
Stop Signal Measures SSRT * HMI_presence	$-$ 0.01	0.007	106.0	$-$ 2.00	0.04
Manchester DBQ Ordinary Violations * HMI_presence	0.47	0.19	106.0	2.44	0.01
Manchester DBQ Errors * HMI_presence	0.95	0.46	106.0	2.03	0.04

### Computational model results: inferring inhibitory control and HMI choice from driving behavior (H3, H4)

Given the various measures collected in the study, we used stepwise regression to select the most important features for training our neural-network based cognitive factor inference model. We combined forward selection, starting with an empty model and adding the predictor that produced the largest increase in model fit, with backward elimination, removing the predictor that produced the smallest decrease in model fit until no further improvement was observed. By following this process, the stepwise regression yielded a set of four cognitive factors to be used in the model: UPPS-P - Positive Urgency, BAS Fun Seeking, goRT_all, and DBQ - Ordinary violations.

**Figure 6:**
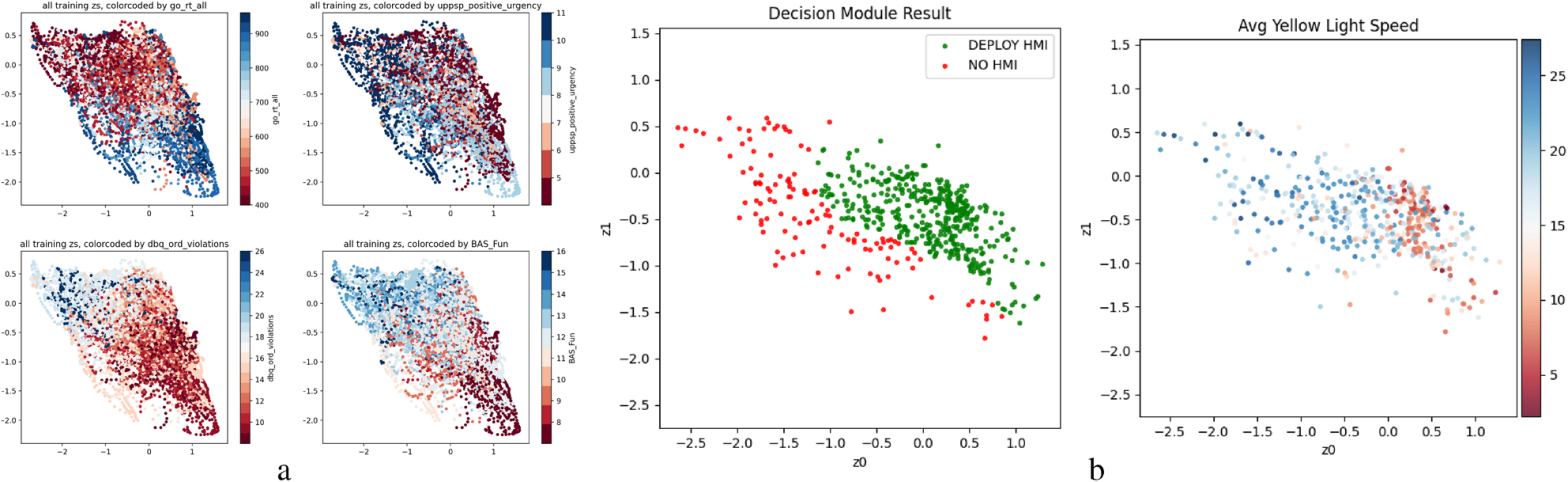
Example embedding and decision module result based on training data from a 27-subject fold; (**a**) Embedding of participants’ past history trajectories with contrastive loss based on four factors: goRT all, UPPS-P Positive Urgency, DBQ Ordinary Violations, and BAS Fun. Colors mark low (red) to high (blue) measures; (**b**) Trained decision boundary (left) and average speed during the yellow light phase conditioned on the decision scheme (right), plotted on the latent embedding space $z_0$, $z_1$. Each point represents a unique time window over which the inference was run.

We adopt the learning approach described to infer cognitive factors based on the subjects’ driving during the experiment. As mentioned earlier, we use the same data to perform training and evaluate model inference. In order to fairly conduct the evaluation, we perform leave-one-out cross-validation over the 27 subjects, averaging model performance over 10 random seeds, and capture properties of the embedding and the resulting training decision criteria performance. We include a complete description of the training and evaluation steps and further findings in the “supplemental information”. The distribution of the inferred latent factors is shown in Fig. 6a. Qualitatively, we observe that fairly strong clustering has emerged for each of the cognitive factors which indicates the effectiveness of the contrastive learning approach is effective. To quantify this further, we show in Table 2 the fit between the distribution of the selected cognitive and the inferred latent factors. Since there is no direct or linear mapping assumed in contrastive learning, we probed the uniformity of the inferred embedding. We used the KL distance between the cognitive measures and the inferred factors’ distribution. The results demonstrate the model’s ability to infer several variables interest centered around impulsivity and inhibitory control.

**Table 2:** Normalized KL Divergences of the subjects for the cognitive measures used in the contrastive loss, averaged over 10 folds (higher is better). We normalize over an ideal clustering result with two Normal distributions separated by a unit-distance (the regularization term $L_3$).

goRT all	UPPS-P Positive Urgency	DBQ Ordinary Violations	BAS Fun Seeking
0.322	0.299	0.288	0.526

BAS Fun is significantly higher, indicating stronger separation.

**Table 3:** Resulting accuracy of interface selection based on the inferred latent factors using test datasets obtained by performing leave-one-out cross-validation on the full set of tests subjects. As can be seen, the inferred latent factors enable personalized HMI selection with 55% balanced accuracy and Cohen’s $\kappa$ = 0.145 compared to a balanced-random HMI choice 50% and $\kappa$ = 0.001, with the personalized scheme reducing yellow light driving speed by 0.59 m/s (standard error=1.58) compared with random.

Decision rule	Mean yellow-light speed (m/s)		Cohen’s Kappa score	Balanced accuracy
	$\mu$	Standard error		
No-HMI	17.36	1.12	0.0	0.50
Always-HMI	15.48	1.10	0.0	0.50
Random	15.69	1.14	0.001	0.50
Window-Averaged (Ours)	**15.10**	**1.09**	**0.145**	**0.56**
Instantaneous (Ours)	15.50	1.10	0.024	0.51

Best performing values are in bold.

We next proceed to probe the efficacy of the resulting latent space to inform HMI adaptation to the subjects. We use leave-one-out to evaluate the decision classifier based on the inferred latent factors. From the test subject’s data, we extract trajectory snippets around the yellow light transitions. The segment of the trajectory before the transition is fed into the context encoder to generate an inferred latent factor. The decision classifier subsequently consumes this latent factor to produce the HMI decision. In order to evaluate the interface selection decisions by the decision classifier we compare them to fixed interface choice chosen optimally for all participants (“one-size-fits-all” approach). We then measure the participants’ behavior in terms of our chosen behavior statistic (mean average speed when yellow light was active) for the selected HMI choice (the classifier’s decision) for the withheld subject averaged over the trials in which the experimental condition matched the decision classifiers output (thereby treating the experiment as a within-subject randomized trial study).

We measure performance of the decision scheme with three metrics: mean yellow light speed, reporting mean ($\mu$) and standard deviation ($\sigma$) aggregated over individuals, along with a Cohen’s $\kappa$ and Balanced Accuracy scores that measure, respectively, accuracy of interface selection scheme under an unbalanced dataset. When leveraging the latent factors to decide on an HMI choice, we achieve a balanced accuracy of 56% and a Cohen Kappa of 0.145 in selecting the optimal HMI for the specific driver, as shown in Table 3, resulting in a reduction of 0.59 m/s in the mean speed throughout the yellow-phase of the traffic light. Additionally, in Fig. 6b (left), we code each of the latents generated from the trajectory snippets according to the decision module’s predictions. In conjunction with Fig. 6b (right), the trajectory snippets for which deployment of the HMI was the decision, we see that the average speed *after* the yellow light transitions is lower, showing the effectiveness of the HMI decision scheme. The color distribution in the different plots demonstrate how the embedding space captures both the driver traits as captured in the questionnaires (a), and the chosen interface decision and resulting driver speed at the yellow light interval (b).

### Limitations

Despite efforts to include a large sample for our study, our sample size was relatively small. Some of this is due to participant motion sickness which at times was quite severe participation had to be ended early. We highlight that this is due to various logistical limitations such as the high costs involved in running a high-fidelity motion simulator study, COVID-related restrictions in recruiting human subjects and the need to implement in-lab social distancing measures, and the technological setup involved with a high-fidelity simulator. We also reiterate that some exclusion of participants was necessary, given our prioritization of a sound dataset over a larger one. While our sample size is in line with what others use in driving simulator studies^67,68^, or machine-learning driving behavior research^69,70^, it is still a relatively small population. We limited our experiment to older and younger participants thinking there would be a larger effect between these two groups. Although this effect did not appear related to age, we found an effect independent of age. Future work should expand the sample to a larger and more representative sample to look at the generalization of these findings. Since our analysis shows promise, a follow-up examining the algorithm’s decisions in real-time would be warranted.

### Conclusion

As traffic accidents and violations frequently occur due to poor impulsivity and inhibitory control, it is important to create driver safety systems that can overcome these cognitive limitations on a personalized level. In this work, we present an approach to infer the individual’s latent factor, the use it to decide when it is or is not appropriate to show a driver safety interface depending on someone’s inferred impulsivity and inhibitory control.

To create this approach, we conducted a driving study using a high-fidelity motion simulator to understand how cognitive factors affect people’s responses to driver safety interfaces. Our study revealed that the prototype interfaces had differing effects on drivers based on their level of impulsivity, as indicated by multiple self-reported and behavioral metrics. In particular, we observed that drivers with lower levels of impulsivity tended to slow down when exposed to the interfaces, while drivers with higher levels of impulsivity exhibited the opposite response. Indeed, previous research has shown that impulsive drivers are more likely to run yellow lights^71^, although yellow lights were designed to warn drivers that they may need to slow down. Our study is the first to show that vehicle safety interfaces may also lead to unintended driving behavior responses for some drivers based on their impulsivity.

Leveraging the data collected in the study, we trained an LSTM network that can infer cognitive traits and, based on these, decide whether or not to employ a driver safety interface. The results show that our decision-making scheme can infer latent factors that are compact, correlate with cognitive measures associated with impulsivity, and can be used effectively to select driver interfaces to improve driver behavior, resulting in lower speed at the zone of dilemma of yellow lights. Although previous work has shown the relationship between cognitive factors such as impulsivity and driving behavior, this is the first time a model is proposed and examined so as to make driver safety recommendations based on cognitive factor inferences conditioned on the driver’s behavior.

The suggested approach lends itself to fleet-scale, online, in-vehicle optimization of the interaction with the driver across the population. If deployed in such a manner, overall improvements in driver safety interfaces may lead to safer roads overall.

### Supplementary Information

Supplementary Figure 1.

Supplementary Figure 2.

Supplementary Figure 3.

Supplementary Figure 4.

Supplementary Figure 5.

Supplementary Figure 6.

Supplementary Figure 7.

## Data Availability

Data and material will be made available upon request by emailing the corresponding authors.
