# Supplementary figures and images for: Personalizing driver safety interfaces via driver cognitive factors inference
Source: Sci Rep. 2024 Aug 5;14:18058. doi: 10.1038/s41598-024-65144-8 (PMC11300826; doi:10.1038/s41598-024-65144-8)

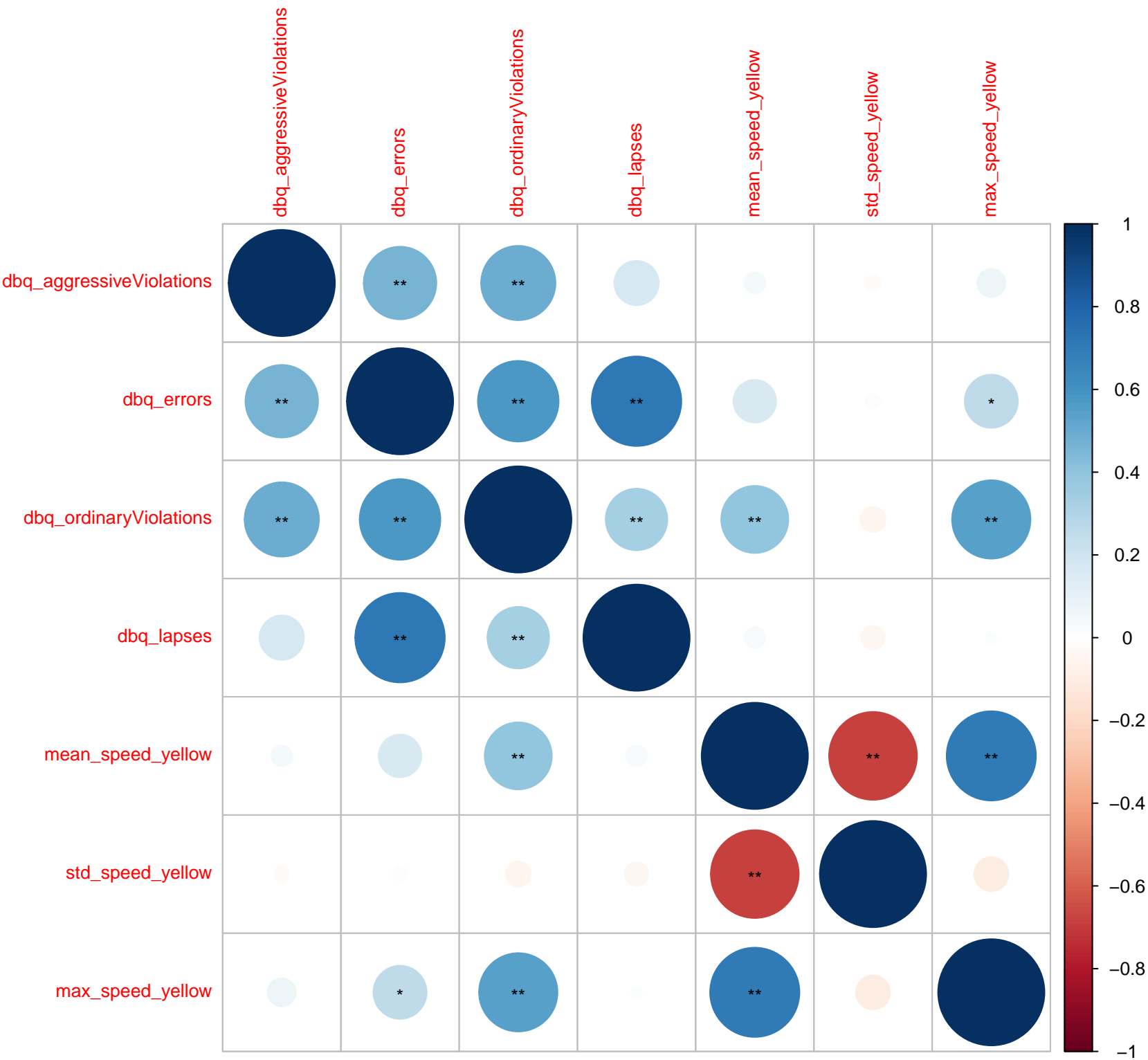

Supplement: Supplementary file 1 — Supplementary Figure 1. [file 41598_2024_65144_MOESM1_ESM.pdf]

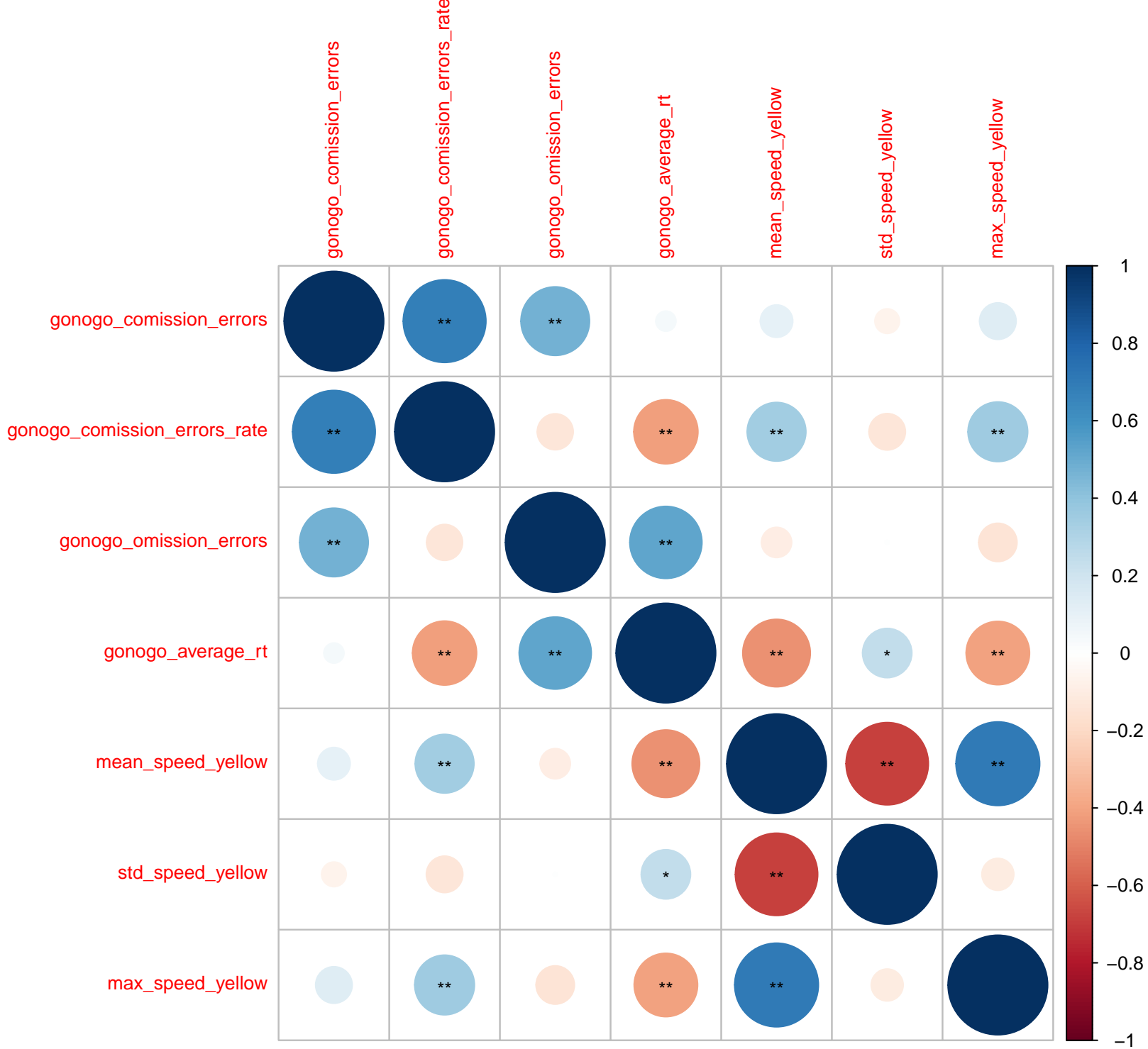

Supplement: Supplementary file 2 — Supplementary Figure 2. [file 41598_2024_65144_MOESM2_ESM.pdf]

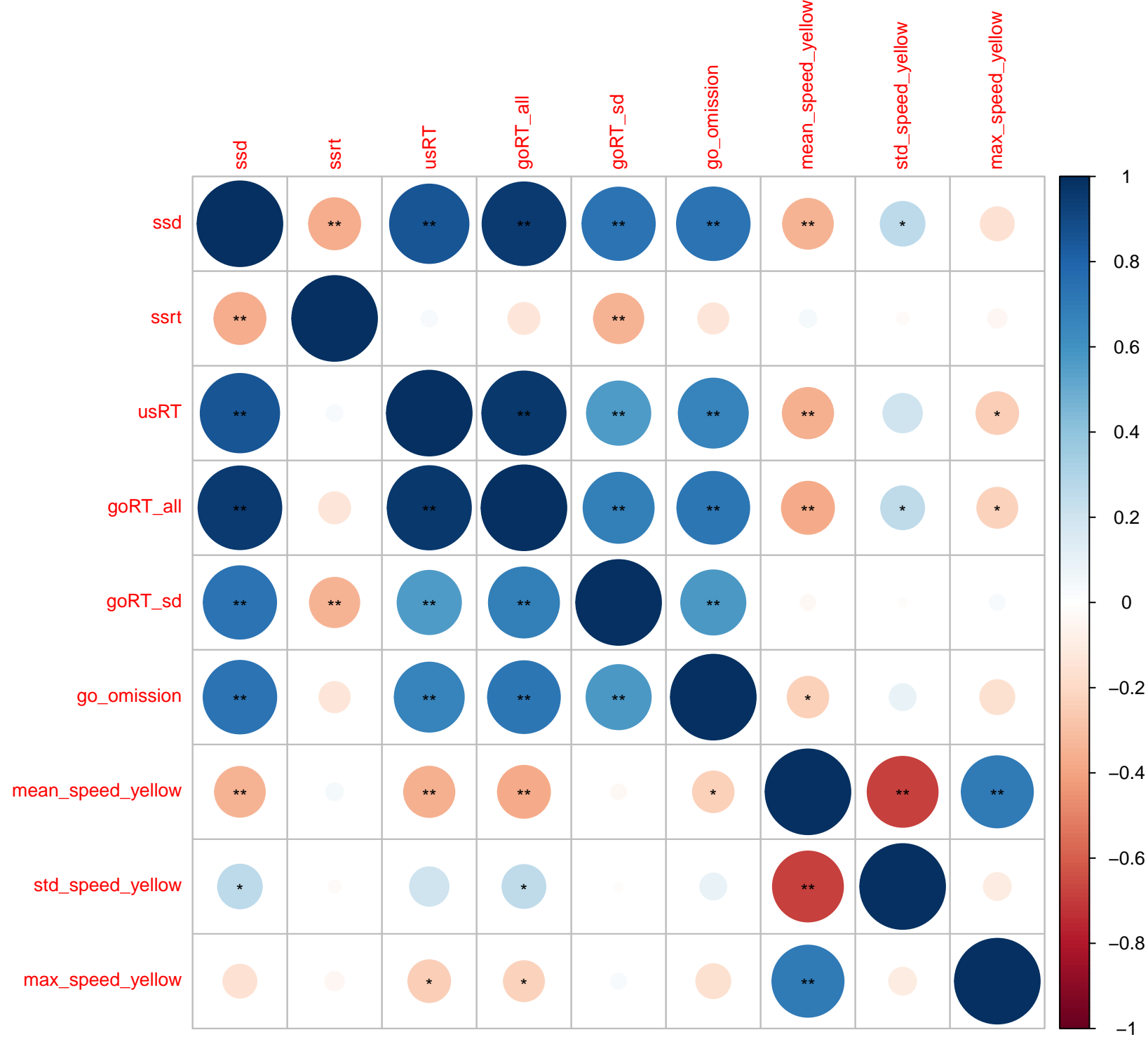

Supplement: Supplementary file 3 — Supplementary Figure 3. [file 41598_2024_65144_MOESM3_ESM.pdf]

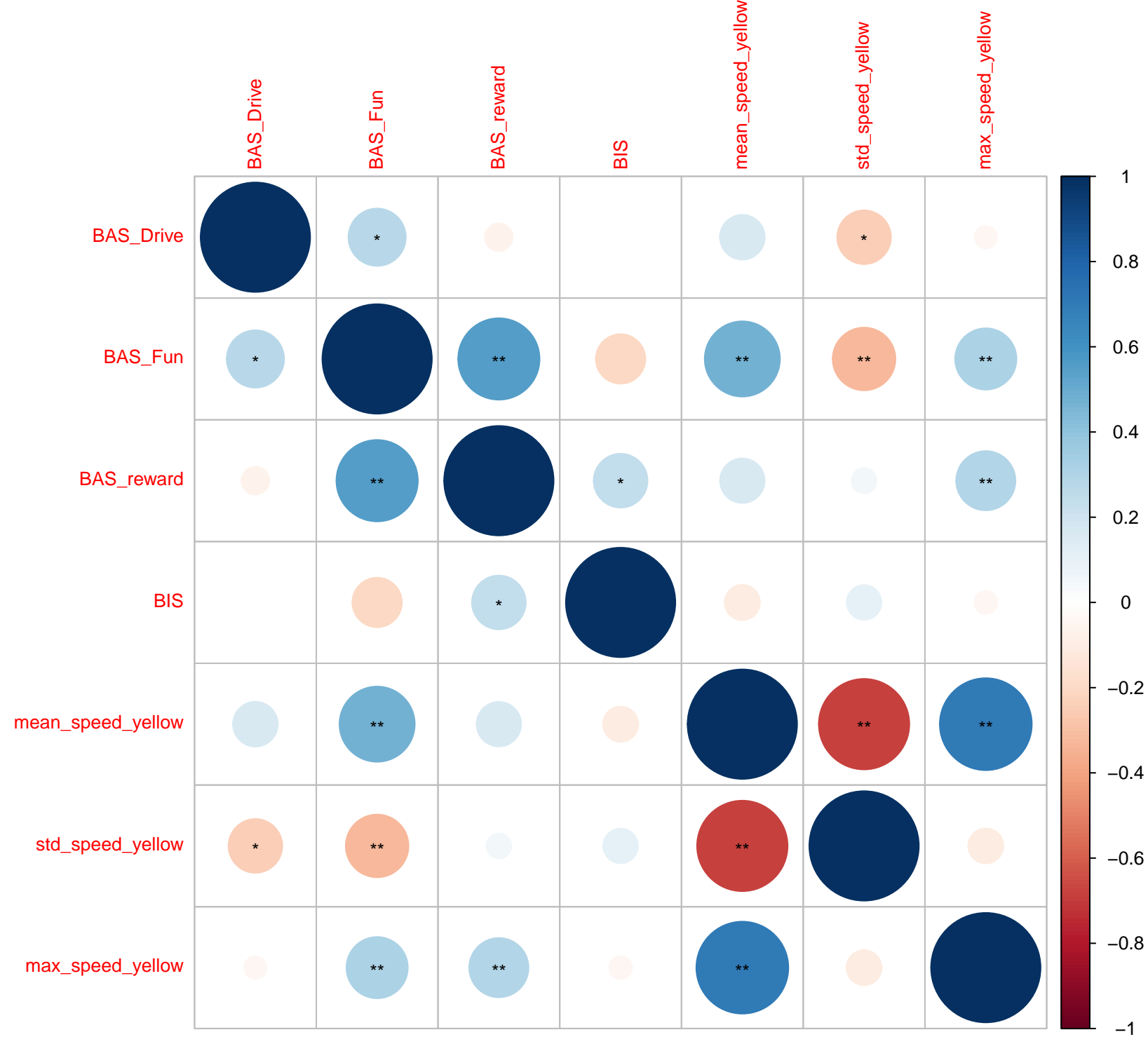

Supplement: Supplementary file 4 — Supplementary Figure 4. [file 41598_2024_65144_MOESM4_ESM.pdf]

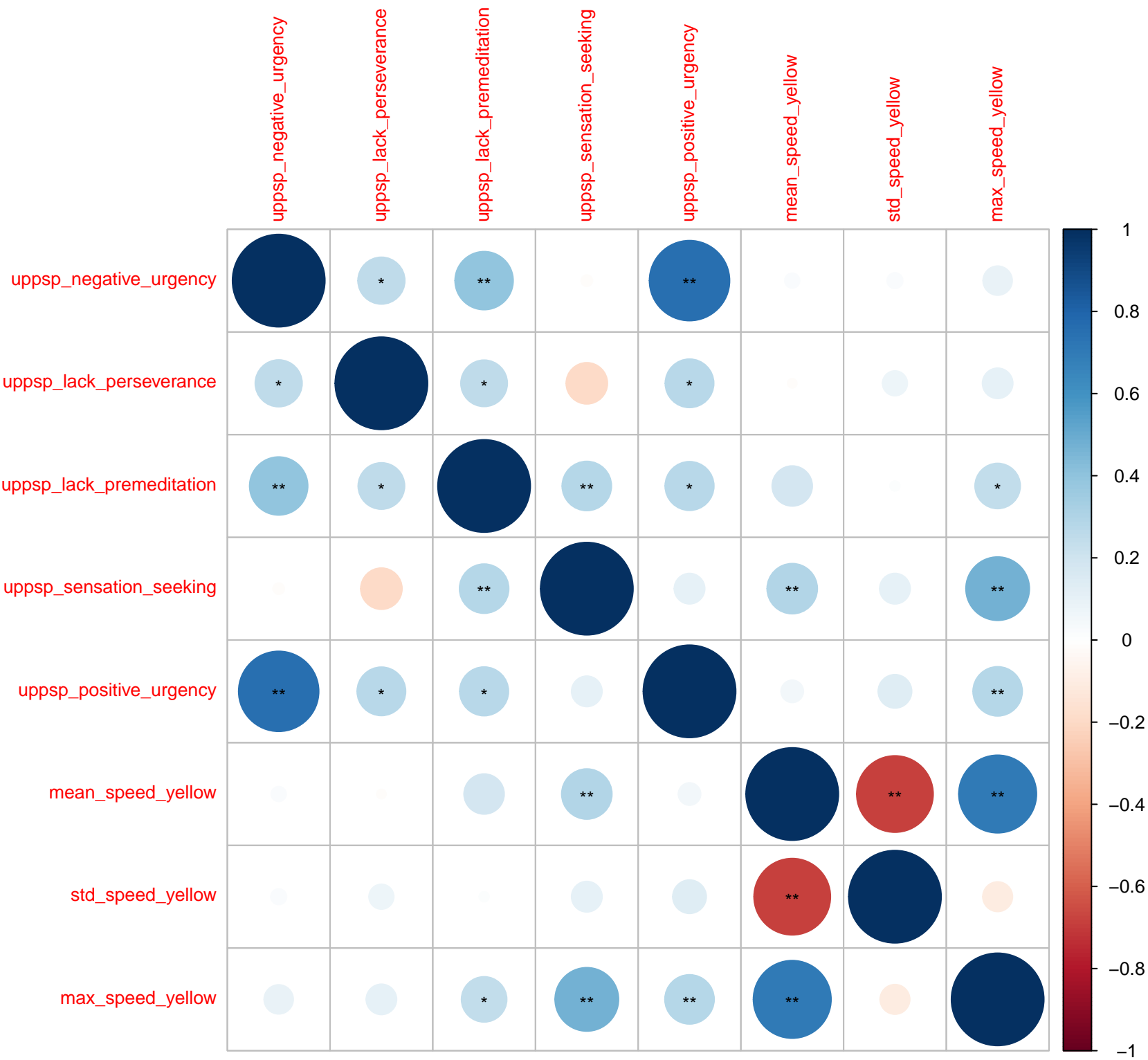

Supplement: Supplementary file 5 — Supplementary Figure 5. [file 41598_2024_65144_MOESM5_ESM.pdf]

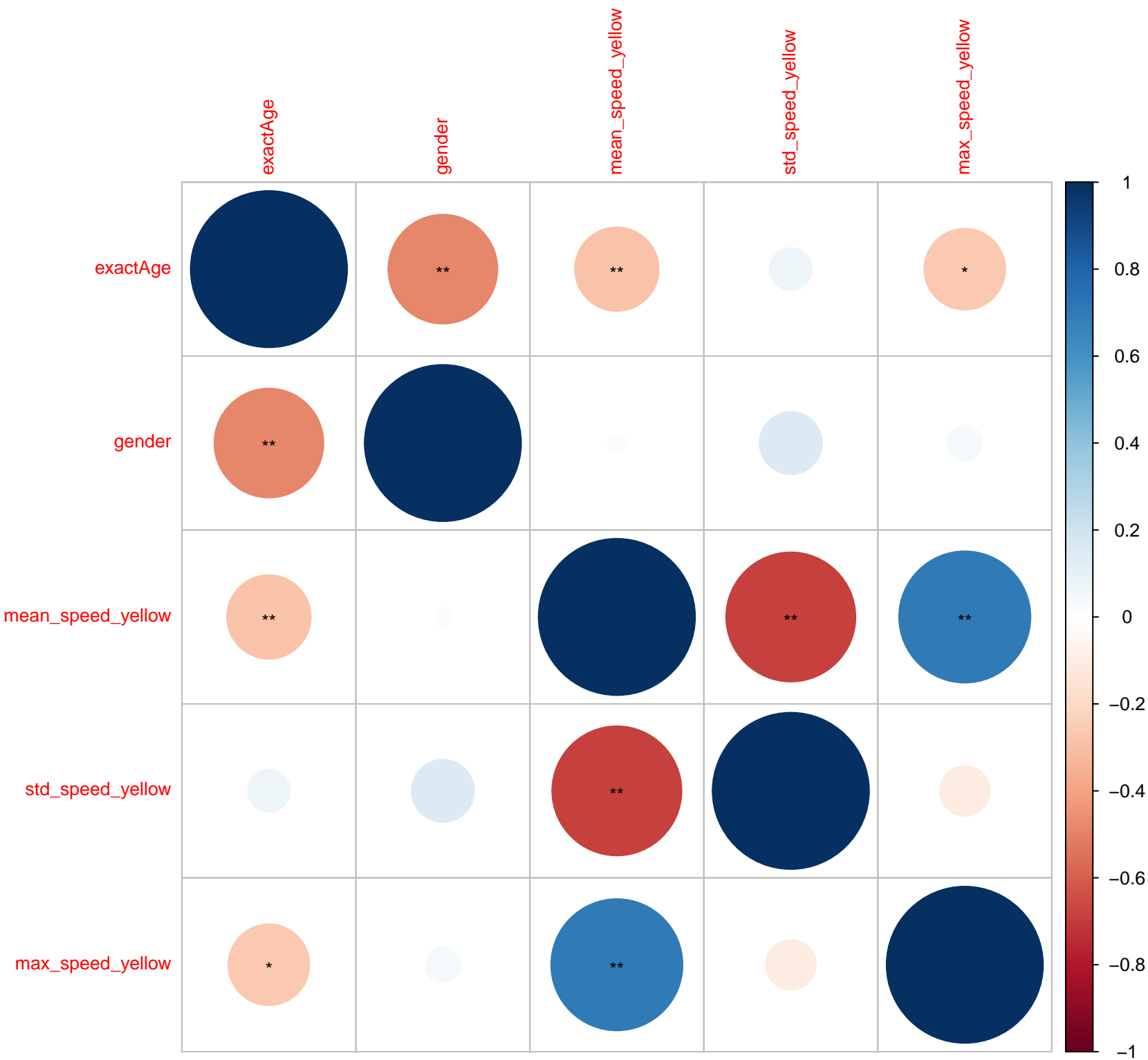

Supplement: Supplementary file 6 — Supplementary Figure 6. [file 41598_2024_65144_MOESM6_ESM.pdf]
