# Supplementary material for: Personalizing driver safety interfaces via driver cognitive factors inference
Source: Sci Rep. 2024 Aug 5;14:18058. doi: 10.1038/s41598-024-65144-8 (PMC11300826; doi:10.1038/s41598-024-65144-8)
Supplement: Supplementary file 7 — Supplementary Figure 7. [file 41598_2024_65144_MOESM7_ESM.pdf]

S1 Supplemental methods

Recruitment

39 external participants were locally recruited through a research partner. Participants were recruited based on their: (1) age, (2) gender, (3) driving experience, and (4) COVID-19 vaccination status. Equal participation based on age groups of interest and gender were used to counterbalance the distribution. Age, ethnicity and race were also taken into consideration during the recruitment process for a more robust and representative sample. Exclusionary criteria in the recruitment process included: (1) inactive driver's license, (2) being pregnant. Participants of interest were contacted by the recruiter for an initial telephone screening. Approved participants would be contacted again by the recruiter and offered an incentive of $150 for the two-hour session. Participants who agreed to participate would then be contacted by email to establish a date and time for their session. Participants would be sent a confirmation email before their session date which would also contain a consent form with explicit instructions of what was to be expected of them.

Protocol

Three participants were scheduled per testing day. Each session lasted approximately two hours. Participants would be greeted by experimental proctors upon arrival and asked to complete a non-disclosure agreement as well as a COVID test before proceeding. Once a negative test result was acquired, along with a completed agreement, the participant was led into the main testing room which included the high fidelity driving simulator and operations station. Upon entering this space, participants were seated on a couch in a sectioned-off intake area. The proctor then provided the participant with a physical version of the consent form they had already signed digiitally. The subject reviewed the form independently while the proctors prepared the software for the simulated driving task; proctors responded to any participant questions at this time. After confirming consent, participants completed a pre-driving survey via Qualtrics. This survey contained demographic questions and short questionnaires (see measures). After completing the surveys, the participant was fitted with a charged E4 Empatica wristband.

S2 Supplemental Results

Effect of the human-machine interfaces on driving behavior

We fitted separate linear mixed models to predict each driving behavior measure based on interface condition. There was a statistically significant of all conditions on the mean speed during the lap, as shown in Figure S1.

Model Details

In this section, we describe in detail how we train and evaluate the context encoder and the decision classifier model.

Training Dataset

In our work τ represents a trajectory of fixed number of samples N of the states, where time is uniformly sampled from $0,\dots,N$ consisting of observations of features and actions made by the driver (e.g. steering, throttling, braking). Specifically, the state (s) consists of a 5-dimensional vector comprised of the following features: longitudinal speed, distance to upcoming intersection start line, distance to upcoming intersection exit line, the state of the upcoming traffic light and whether the HMI was active or not. The action (a) consists of the longitudinal acceleration.

The dataset we use for context encoder training consists of trajectory snippets ($\sim$22k) extracted around green to yellow light traffic light transitions (with window size equal to the context length used by the LSTM and a hopsize of 1) from all (baseline and HMI) laps from all the subjects.

For the context encoder we use a history of driving behavior (temporally extended state and actions pairs) as inputs to train a long-term short-memory (LSTM) encoder. LSTMs have been shown to produce strong results in learning latent characteristics of drivers based on driving data and supervisory signals, such as level of aggression, safety, etc.[1]. Hyperparameter details are presented in Table S1. The encoder structure consists of a single hidden layer LSTM with 128 units that maps the state and action history to a 2-dimensional latent space. The LSTM uses a context length of 30 timesteps which amounts to 6 seconds of driving behavior at 5 Hz. The hidden state of the LSTM is mapped to the mean and log of the standard deviation of the latent space. During each network training update, we conduct a forward pass using a batch (batch size = 2048) of training samples of past driving history and cognitive measures followed by network parameter update via backpropagation using the Adam optimizer. We select loss coefficients, batch size, and training epochs to empirically achieve reasonable convergence in the overall loss without overfitting.

Decision Classifier

Our decision model uses an ε-Support Vector Regression model with a polynomial kernel of degree 3 to regress over the continuous-valued training targets. The continuous-valued training target for each sample z corresponds to the difference in the mean speeds when yellow lights are active between laps with and without HMI for that subject. Classification is done as a

Table S1. Hyperparameters used in the training experiments.

Hyperparameter	Value	Hyperparameter	Value
Batch size	2048	Latent dimension	2
Number of training epochs	600	Reconstruction loss coefficient α_1	10^4
Contrastive margin ε	2	Contrastive loss coefficient α_2	10^4
Learning rate	10^{-2}	Latent regularizer loss coefficient α_3	10^{-8}
LSTM context length	6 s	Support vector regression polynomial degree	3
LSTM hidden layers	1	Support vector regression margin ε_{SVR}	0.5
LSTM hidden size	128		

seprate step, with the decision threshold set to zero. The input to the decision model is the two-dimensional latent vector, z generated by the trained context encoder on a dataset that only consists of trajectory snippets just preceding a green-to-yellow transition.

For the main reported results, we train the decision model only on latent vectors generated using the pre-trained context encoder from trajectory snippets that lead up to green to yellow traffic lights transitions sampled from all the laps. We filtered out snippets in which the subject was only exposed to an upcoming yellow light for less than 1s. The final dataset consists of 555 snippets extracted from 135 laps driven by 27 subjects. Out of the 555 snippets, 464 are from laps in which HMIs were deployed and the remaining 91 were from non-HMI laps.

Latent Factor Inference Modes

The results in Table 3 included two schemes chosen for processing z's as inputs to the decision classifier, both in training and inference, which we now expand upon. For the *Instantaneous* case shown in Table 3, the entire trajectory snippet is passed through the LSTM encoder and we consider z to be obtained from the hidden state at only the last time step of the snippet. We also explored a *Windowed-Average* case, which we hypothesize better captures globally stable properties (for example, cognitive measures) of a particular driver. To do this, we perform temporal averaging of the z's generated at every timestep in the snippet over the entirety of the snippet. For each training configuration, we train the model 10 times using different random seeds and report the averaged results.

Effect of Sequential Inference on Model Performance

To evaluate the performance of the context encoder and decision classifier as it might be deployed in a real world setting with streaming data, we execute the decision maker using all data fed sequentially into the model. Note that the LSTM context encoder is a recurrent model, so it will aggregate an estimate of z in a way that is dependent on the history of driving behavior. In Table S2, we observe similar trends to Table 3 and that our system outperforms the baselines, however, the improvement is milder. In particular, Table 3 showed 0.59 m/s (standard error=1.58) reduction in yellow-light speed using Windowed-Average scheme, though when using streaming data, this reduction drops to 0.23 m/s (standard error=1.52) when comparing the same decision rules. It is hypothesized that this drop in speed reduction may have occurred due to the noise introduced when using streaming data, and the fact that the LSTM retained state from data outside of the training context (e.g. periods of the drive when the lights were not visible to the driver). Nonetheless, we can see that the inferred latent factors enable personalized HMI selection with 55% accuracy, resulting in a mean yellow light speed of 15.23 m/s and decrease of 0.24 m/s compared to the globally-optimal Always-HMI case.

References

1. Morton, J. & Kochenderfer, M. J. Simultaneous policy learning and latent state inference for imitating driver behavior. In *2017 IEEE 20th International Conference on Intelligent Transportation Systems (ITSC)*, 1–6 (2017).

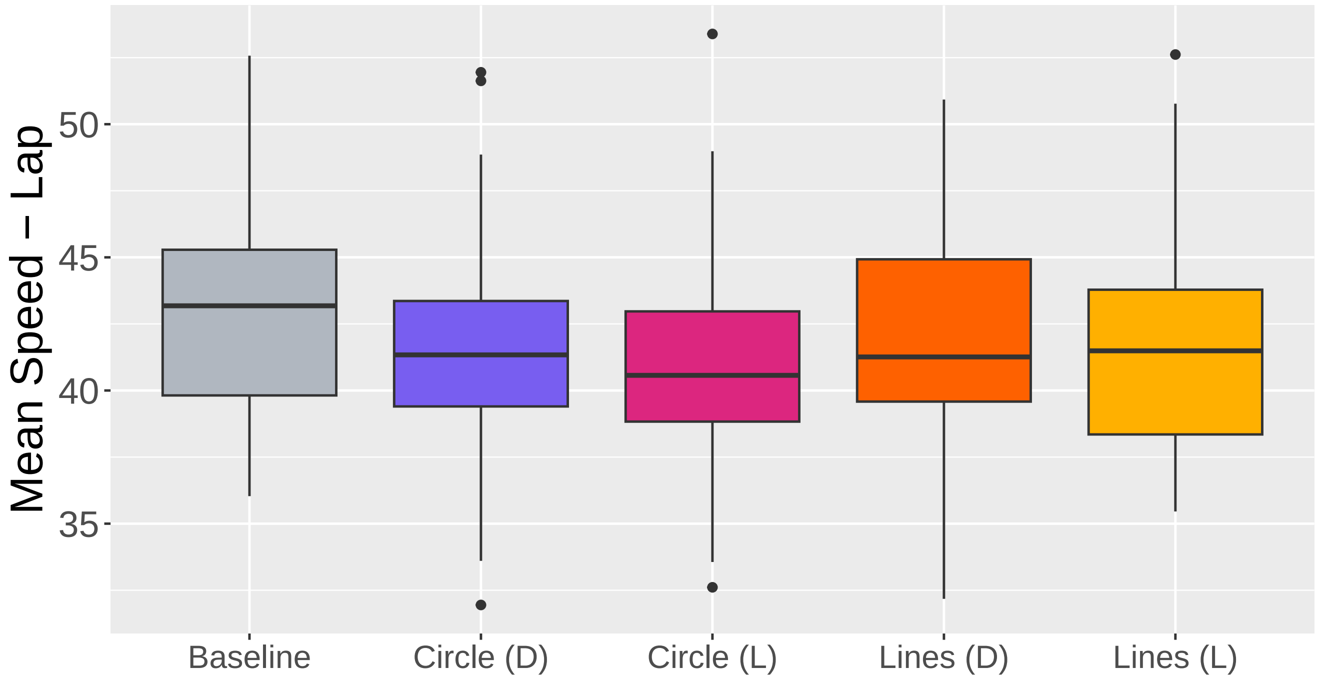

Figure S1. Mean speed during the lap in each condition. In all laps with HMI, participants had a lower mean speed during the lap in comparison to the baseline lap

Table S2. Resulting accuracy of interface selection based on sequentially predicted latent factors on streaming data, averaged over 5 random seeds for the testing set over 27 folds. We use a moving average filter (of window length of 6s) to smooth out the sequential latent factor estimate time series. Last two rows of show results from an ablation in which the reconstruction loss was set to be 0.0

Decision Rule	Mean Yellow-Light Speed (m/s)		Cohen's Kappa Score	Balanced Accuracy
	μ	Standard Error		
No-HMI	17.36	1.12	0.0	0.50
Always-HMI	15.48	1.10	0.0	0.50
Random	15.47	1.08	-0.036	0.47
Window-Averaged (Ours) - $\alpha_1 = 10^4$	15.28	1.09	0.086	0.54
Instantaneous (Ours) - $\alpha_1 = 10^4$	15.46	1.10	0.006	0.50
Window-Averaged (Ours) - $\alpha_1 = 0.0$	**15.24**	**1.07**	**0.108**	**0.55**
Instantaneous (Ours) - $\alpha_1 = 0.0$	15.46	1.09	0.071	0.52

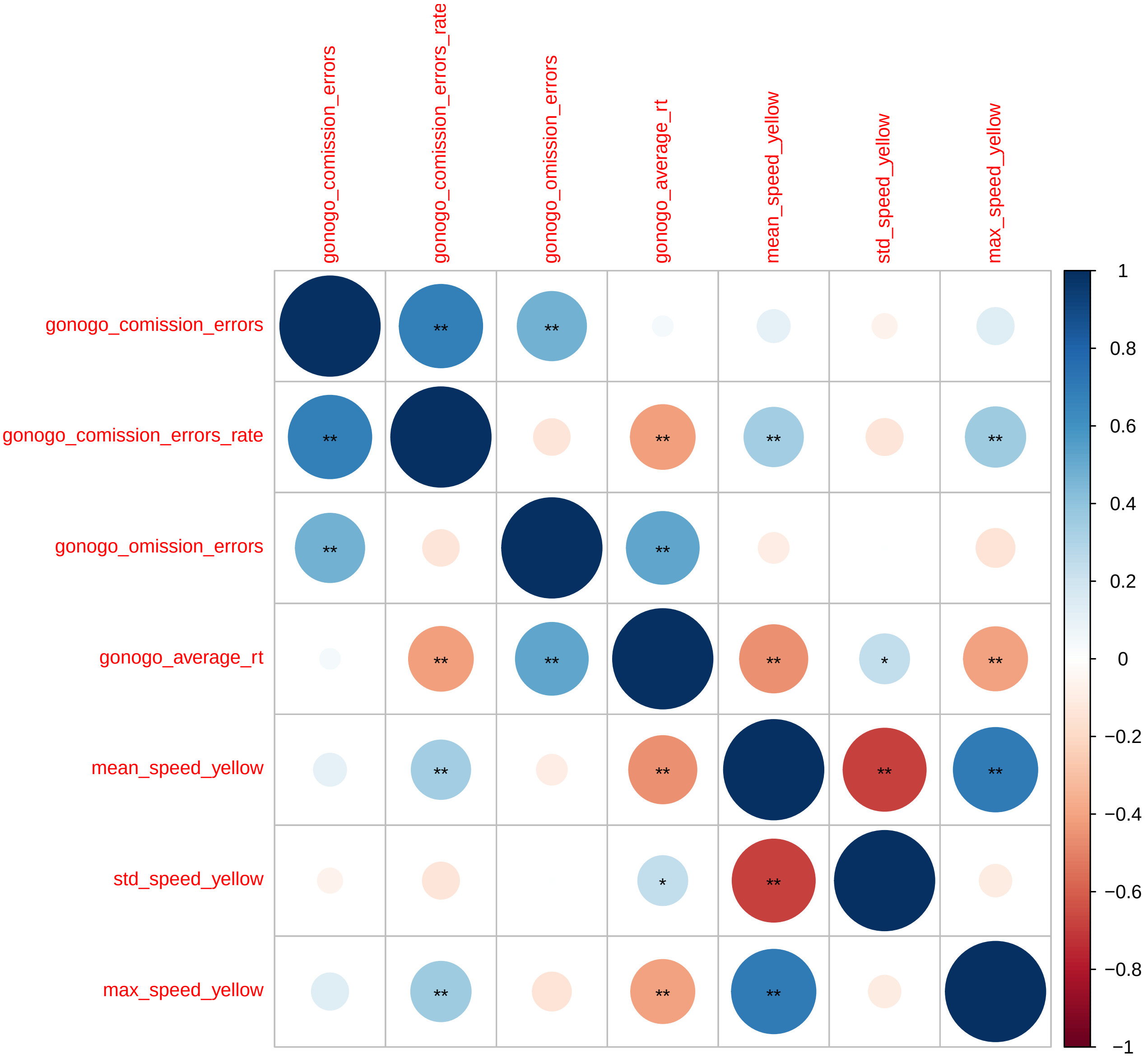

Figure S2. Correlation Matrix of Go/No-Go and Speed Measures

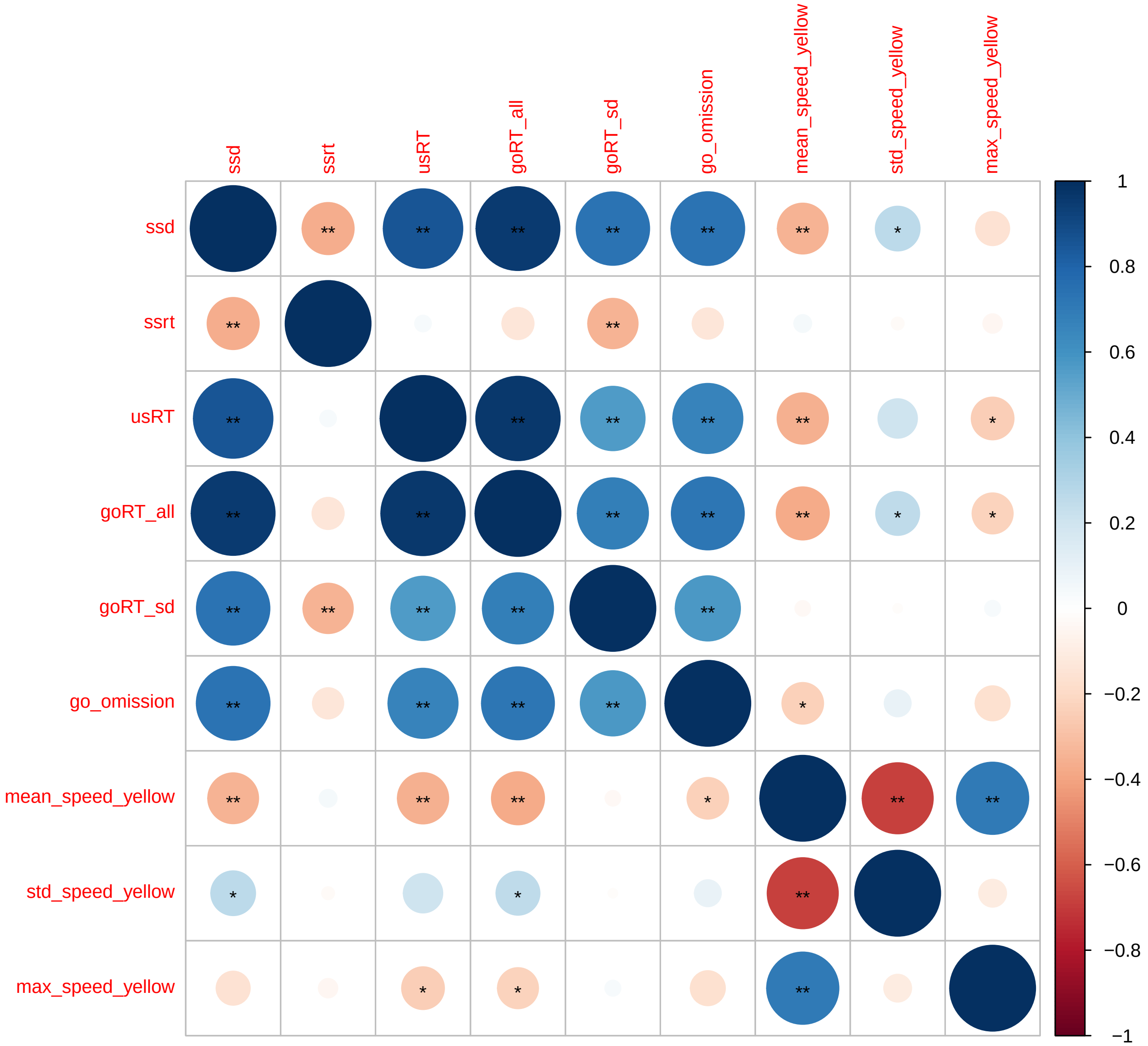

Figure S3. Correlation Matrix of Stop Signal and Speed Measures

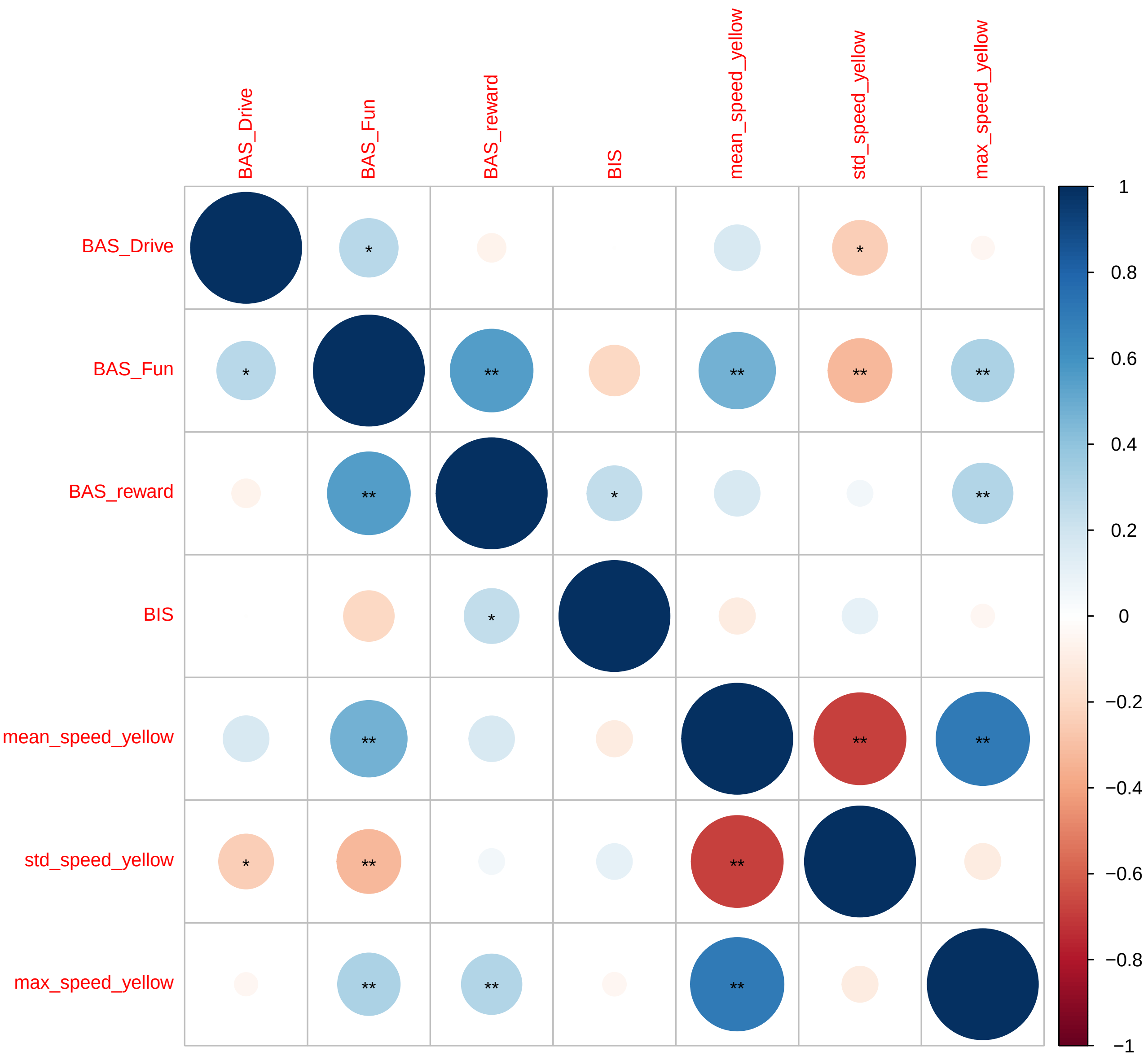

Figure S4. Correlation Matrix of BIS/BAS and Speed Measures

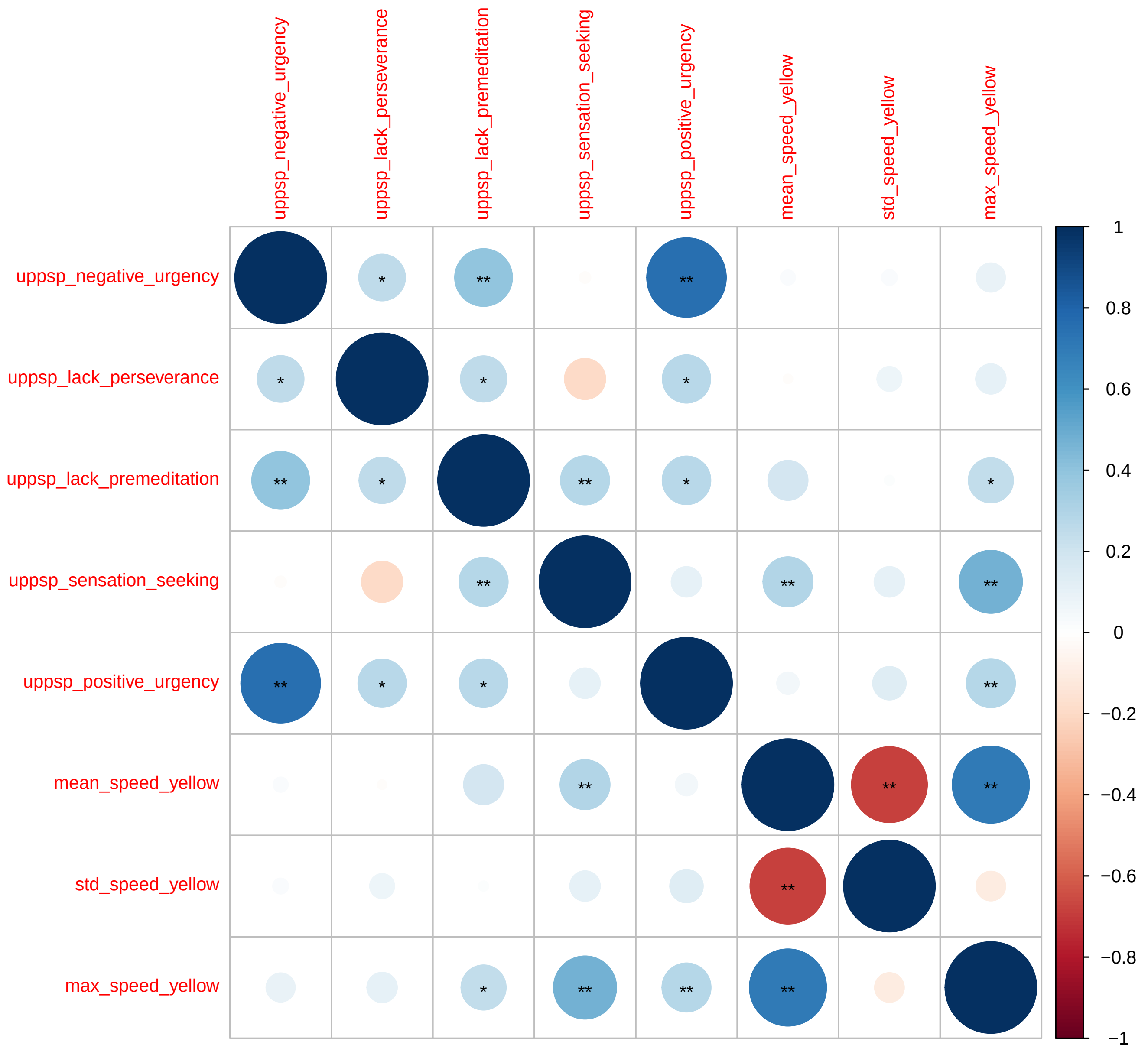

Figure S5. Correlation Matrix of UPPS-P and Speed Measures

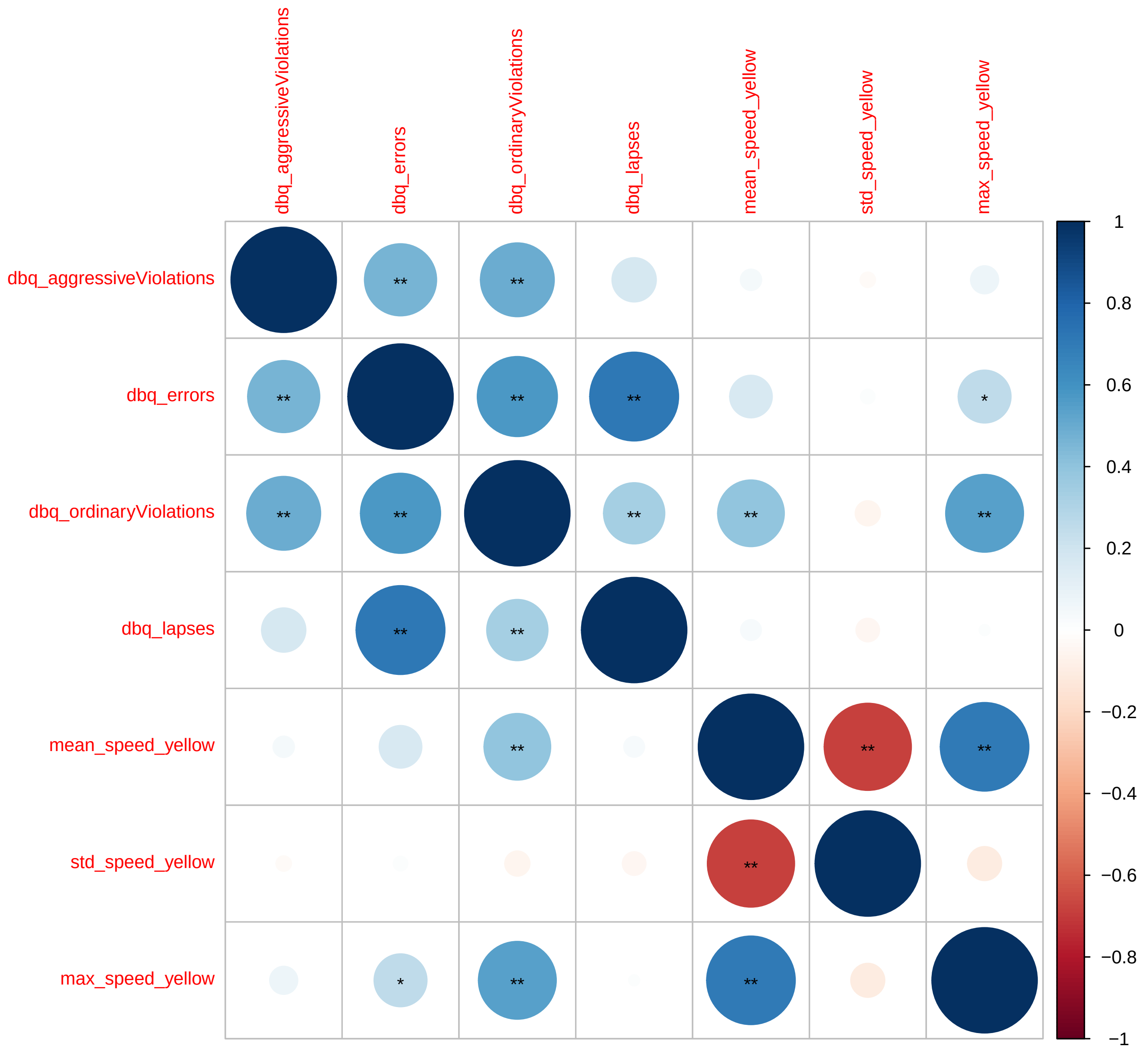

Figure S6. Correlation Matrix of Manchester DBQ and Speed Measures

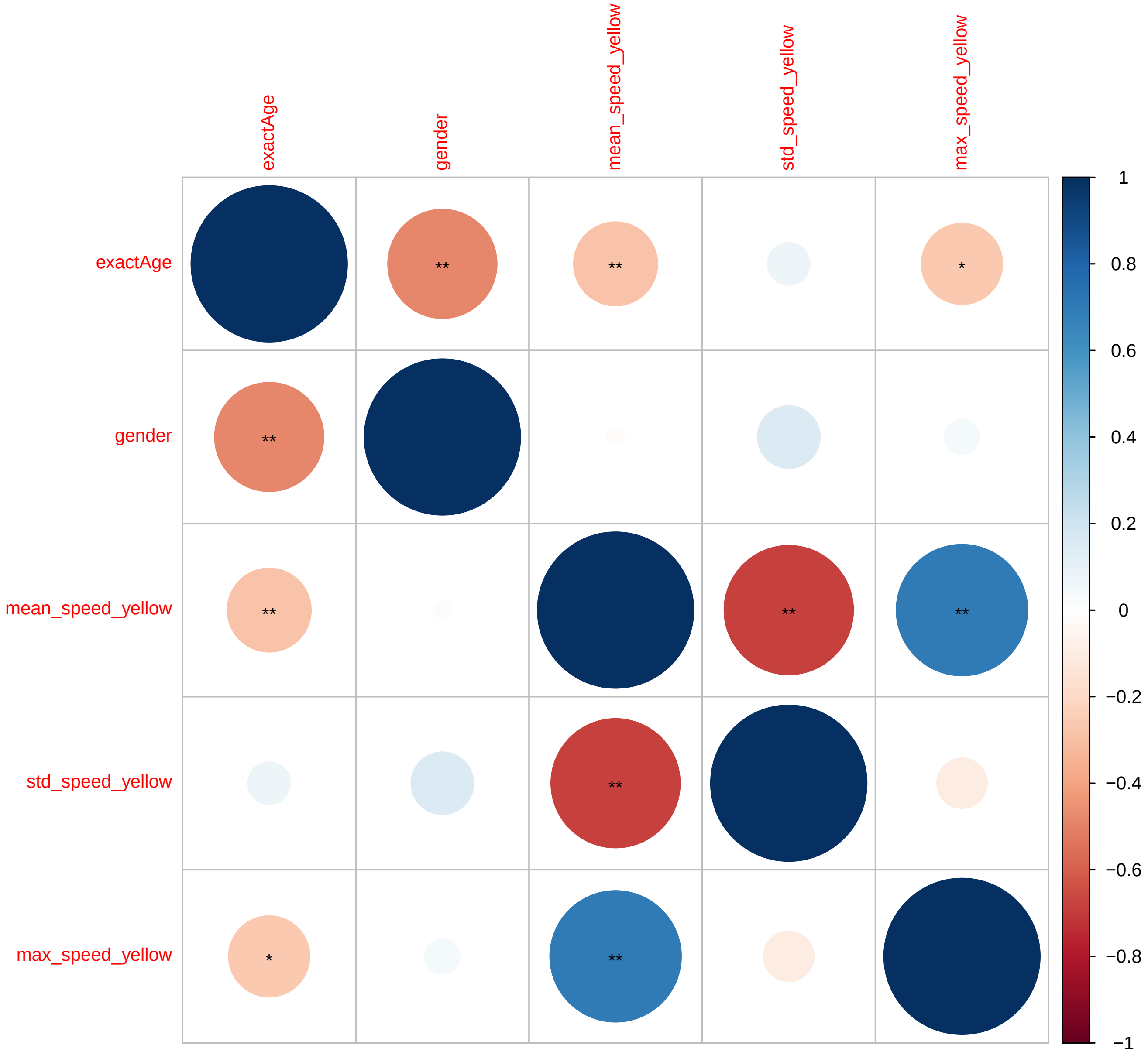

Figure S7. Correlation Matrix of Demographics and Speed Measures

Table S3. Go/No-Go Measures - Bayesian Pearson Correlations

			Pearson's r	BF_{10}	Lower 95% CI	Upper 95% CI
gonogo_comission_errors	-	gonogo_comission_errors_rate	$0.690***$	$5.060 \times 10^{+16}$	0.582	0.767
	-	gonogo_omission_errors	$0.475***$	$1.078 \times 10^{+6}$	0.325	0.594
	-	gonogo_average_rt	0.041	0.122	-0.131	0.210
	-	mean_speed_yellow	0.107	0.225	-0.066	0.271
	-	std_speed_yellow	-0.062	0.139	-0.229	0.111
	-	max_speed_yellow	0.135	0.352	-0.037	0.297
	-	min_speed_yellow	0.071	0.151	-0.101	0.238
gonogo_comission_errors_rate	-	gonogo_omission_errors	-0.131	0.326	-0.293	0.042
	-	gonogo_average_rt	$-0.414***$	15714.643	-0.543	-0.257
	-	mean_speed_yellow	$0.348***$	375.156	0.184	0.487
	-	std_speed_yellow	-0.134	0.347	-0.297	0.038
	-	max_speed_yellow	$0.360***$	681.940	0.197	0.497
	-	min_speed_yellow	0.172	0.727	-4.975×10^{-4}	0.331
gonogo_omission_errors	-	gonogo_average_rt	$0.525***$	$7.110 \times 10^{+7}$	0.383	0.635
	-	mean_speed_yellow	-0.093	0.189	-0.258	0.080
	-	std_speed_yellow	0.002	0.110	-0.169	0.172
	-	max_speed_yellow	-0.148	0.446	-0.309	0.024
	-	min_speed_yellow	-0.032	0.117	-0.201	0.140
gonogo_average_rt	-	mean_speed_yellow	$-0.460***$	352747.434	-0.581	-0.309
	-	std_speed_yellow	0.245	5.482	0.075	0.397
	-	max_speed_yellow	$-0.406***$	9205.034	-0.536	-0.248
	-	min_speed_yellow	$-0.310**$	63.828	-0.454	-0.143
mean_speed_yellow	-	std_speed_yellow	$-0.684***$	$9.245 \times 10^{+16}$	-0.761	-0.577
	-	max_speed_yellow	$0.706***$	$4.952 \times 10^{+18}$	0.605	0.779
	-	min_speed_yellow	$0.809***$	$1.845 \times 10^{+29}$	0.737	0.858
std_speed_yellow	-	max_speed_yellow	-0.103	0.218	-0.265	0.066
	-	min_speed_yellow	$-0.911***$	$2.642 \times 10^{+49}$	-0.935	-0.874
max_speed_yellow	-	min_speed_yellow	$0.362***$	1081.461	0.203	0.496

* $BF_{10} > 10$, ** $BF_{10} > 30$, *** $BF_{10} > 100$

Table S4. Stop Signal Measures - Bayesian Pearson Correlations

			Pearson's r	BF_{10}	Lower 95% CI	Upper 95% CI
ssd	-	ssrt	$-0.366***$	1347.018	-0.500	-0.207
	-	usRT	$0.857***$	$6.229 \times 10^{+36}$	0.801	0.895
	-	goRT_all	$0.954***$	$3.339 \times 10^{+67}$	0.934	0.966
	-	goRT_sd	$0.730***$	$5.189 \times 10^{+20}$	0.635	0.798
	-	go_omission	$0.736***$	$1.628 \times 10^{+21}$	0.642	0.802
	-	mean_speed_yellow	$-0.349***$	524.864	-0.485	-0.188
	-	std_speed_yellow	$0.269*$	15.043	0.104	0.415
	-	max_speed_yellow	-0.155	0.533	-0.313	0.014
	-	min_speed_yellow	$-0.274*$	18.012	-0.420	-0.109
ssrt	-	usRT	0.036	0.117	-0.132	0.202
	-	goRT_all	-0.138	0.381	-0.297	0.031
	-	goRT_sd	$-0.342***$	366.723	-0.479	-0.181
	-	go_omission	-0.131	0.333	-0.290	0.039
	-	mean_speed_yellow	0.042	0.121	-0.127	0.207
	-	std_speed_yellow	-0.021	0.111	-0.188	0.147
	-	max_speed_yellow	-0.050	0.127	-0.215	0.119
	-	min_speed_yellow	-1.106×10^{-4}	0.108	-0.167	0.167
usRT	-	goRT_all	$0.965***$	$3.003 \times 10^{+75}$	0.950	0.975
	-	goRT_sd	$0.560***$	$5.786 \times 10^{+9}$	0.428	0.662
	-	go_omission	$0.665***$	$4.605 \times 10^{+15}$	0.554	0.747
	-	mean_speed_yellow	$-0.355***$	731.749	-0.490	-0.195
	-	std_speed_yellow	0.209	1.999	0.041	0.361
	-	max_speed_yellow	-0.246	6.393	-0.394	-0.079
	-	min_speed_yellow	-0.232	4.076	-0.382	-0.065
goRT_all	-	goRT_sd	$0.686***$	$1.251 \times 10^{+17}$	0.579	0.763
	-	go_omission	$0.720***$	$7.035 \times 10^{+19}$	0.623	0.790
	-	mean_speed_yellow	$-0.380***$	2933.574	-0.512	-0.222
	-	std_speed_yellow	0.260	10.406	0.093	0.407
	-	max_speed_yellow	-0.226	3.384	-0.377	-0.059
	-	min_speed_yellow	-0.277	19.944	-0.422	-0.111
goRT_sd	-	go_omission	$0.576***$	$3.075 \times 10^{+10}$	0.446	0.675
	-	mean_speed_yellow	-0.032	0.115	-0.198	0.136
	-	std_speed_yellow	-0.012	0.109	-0.178	0.156
	-	max_speed_yellow	0.033	0.116	-0.135	0.199
	-	min_speed_yellow	0.042	0.121	-0.127	0.207
go_omission	-	mean_speed_yellow	-0.233	4.179	-0.383	-0.066
	-	std_speed_yellow	0.097	0.199	-0.073	0.259
	-	max_speed_yellow	-0.164	0.645	-0.321	0.005
	-	min_speed_yellow	-0.070	0.148	-0.233	0.100
mean_speed_yellow	-	std_speed_yellow	$-0.684***$	$9.245 \times 10^{+16}$	-0.761	-0.577
	-	max_speed_yellow	$0.706***$	$4.952 \times 10^{+18}$	0.605	0.779
	-	min_speed_yellow	$0.809***$	$1.845 \times 10^{+29}$	0.737	0.858
std_speed_yellow	-	max_speed_yellow	-0.103	0.218	-0.265	0.066
	-	min_speed_yellow	$-0.911***$	$2.642 \times 10^{+49}$	-0.935	-0.874
max_speed_yellow	-	min_speed_yellow	$0.362***$	1081.461	0.203	0.496

* $BF_{10} > 10$, ** $BF_{10} > 30$, *** $BF_{10} > 100$

Table S5. BIS/BAS Measures - Bayesian Pearson Correlations

			Pearson's r	BF_{10}	Lower 95% CI	Upper 95% CI
BAS_Drive	-	BAS_Fun	$0.275*$	18.825	0.110	0.421
	-	BAS_reward	-0.065	0.142	-0.229	0.104
	-	BIS	-3.527×10^{-4}	0.108	-0.168	0.167
	-	mean_speed_yellow	0.168	0.709	-7.937×10^{-4}	0.325
	-	std_speed_yellow	-0.242	5.589	-0.391	-0.075
	-	max_speed_yellow	-0.043	0.121	-0.208	0.126
	-	min_speed_yellow	0.220	2.770	0.052	0.371
BAS_Fun	-	BAS_reward	$0.554***$	$2.848\times10^{+9}$	0.420	0.657
	-	BIS	-0.206	1.823	-0.358	-0.037
	-	mean_speed_yellow	$0.473***$	$1.700\times10^{+6}$	0.326	0.590
	-	std_speed_yellow	$-0.329***$	196.005	-0.468	-0.167
	-	max_speed_yellow	$0.315**$	99.190	0.152	0.455
	-	min_speed_yellow	$0.362***$	1075.032	0.203	0.496
BAS_reward	-	BIS	0.242	5.668	0.075	0.391
	-	mean_speed_yellow	0.167	0.681	-0.003	0.323
	-	std_speed_yellow	0.053	0.129	-0.116	0.217
	-	max_speed_yellow	$0.294**$	39.632	0.129	0.437
	-	min_speed_yellow	0.038	0.118	-0.131	0.203
BIS	-	mean_speed_yellow	-0.104	0.221	-0.266	0.065
	-	std_speed_yellow	0.102	0.212	-0.068	0.263
	-	max_speed_yellow	-0.044	0.122	-0.209	0.125
	-	min_speed_yellow	-0.110	0.240	-0.271	0.059
mean_speed_yellow	-	std_speed_yellow	$-0.684***$	$9.245\times10^{+16}$	-0.761	-0.577
	-	max_speed_yellow	$0.706***$	$4.952\times10^{+18}$	0.605	0.779
	-	min_speed_yellow	$0.809***$	$1.845\times10^{+29}$	0.737	0.858
std_speed_yellow	-	max_speed_yellow	-0.103	0.218	-0.265	0.066
	-	min_speed_yellow	$-0.911***$	$2.642\times10^{+49}$	-0.935	-0.874
max_speed_yellow	-	min_speed_yellow	$0.362***$	1081.461	0.203	0.496

* $BF_{10} > 10$, ** $BF_{10} > 30$, *** $BF_{10} > 100$

Table S6. UPPS-P Bayesian Pearson Correlations

			Pearson's r	BF_{10}	Lower 95% CI	Upper 95% CI
uppsp_negative_urgency	-	uppsp_lack_perseverance	0.257	9.623	0.091	0.405
	-	uppsp_lack_premeditation	0.396 ***	7487.515	0.240	0.525
	-	uppsp_sensation_seeking	−0.015	0.109	−0.181	0.153
	-	uppsp_positive_urgency	0.751 ***	$5.046 \times 10^{+22}$	0.662	0.814
	-	mean_speed_yellow	0.026	0.113	−0.142	0.192
	-	std_speed_yellow	0.029	0.114	−0.139	0.195
	-	max_speed_yellow	0.100	0.207	−0.070	0.261
	-	min_speed_yellow	−0.079	0.163	−0.243	0.090
uppsp_lack_perseverance	-	uppsp_lack_premeditation	0.253	8.075	0.086	0.400
	-	uppsp_sensation_seeking	−0.200	1.544	−0.353	−0.031
	-	uppsp_positive_urgency	0.275*	18.855	0.110	0.421
	-	mean_speed_yellow	−0.010	0.108	−0.177	0.157
	-	std_speed_yellow	0.073	0.152	−0.097	0.236
	-	max_speed_yellow	0.108	0.233	−0.061	0.269
	-	min_speed_yellow	−0.105	0.224	−0.267	0.064
uppsp_lack_premeditation	-	uppsp_sensation_seeking	0.284*	26.998	0.119	0.429
	-	uppsp_positive_urgency	0.279*	21.447	0.113	0.424
	-	mean_speed_yellow	0.189	1.158	0.020	0.343
	-	std_speed_yellow	0.012	0.109	−0.156	0.178
	-	max_speed_yellow	0.240	5.282	0.073	0.389
	-	min_speed_yellow	−0.021	0.111	−0.187	0.147
uppsp_sensation_seeking	-	uppsp_positive_urgency	0.109	0.236	−0.060	0.270
	-	mean_speed_yellow	0.295 **	42.896	0.131	0.438
	-	std_speed_yellow	0.110	0.237	−0.060	0.271
	-	max_speed_yellow	0.471 ***	$1.540 \times 10^{+6}$	0.325	0.589
	-	min_speed_yellow	0.024	0.112	−0.144	0.190
uppsp_positive_urgency	-	mean_speed_yellow	0.059	0.135	−0.110	0.223
	-	std_speed_yellow	0.131	0.337	−0.038	0.291
	-	max_speed_yellow	0.284*	26.932	0.119	0.429
	-	min_speed_yellow	−0.116	0.261	−0.277	0.054
mean_speed_yellow	-	std_speed_yellow	−0.684 ***	$9.245 \times 10^{+16}$	−0.761	−0.577
	-	max_speed_yellow	0.706 ***	$4.952 \times 10^{+18}$	0.605	0.779
	-	min_speed_yellow	0.809 ***	$1.845 \times 10^{+29}$	0.737	0.858
std_speed_yellow	-	max_speed_yellow	−0.103	0.218	−0.265	0.066
	-	min_speed_yellow	−0.911 ***	$2.642 \times 10^{+49}$	−0.935	−0.874
max_speed_yellow	-	min_speed_yellow	0.362 ***	1081.461	0.203	0.496

* $BF_{10} > 10$, ** $BF_{10} > 30$, *** $BF_{10} > 100$

Table S7. Manchester DBQ Measures - Bayesian Pearson Correlations

			Pearson's r	BF_{10}	Lower 95% CI	Upper 95% CI
dbq_aggressiveViolations	-	dbq_errors	0.468 ∗∗∗	$1.139 \times 10^{+6}$	0.320	0.586
	-	dbq_ordinaryViolations	0.490 ∗∗∗	$7.218 \times 10^{+6}$	0.346	0.605
	-	dbq_lapses	0.176	0.844	0.007	0.332
	-	mean_speed_yellow	0.040	0.120	−0.128	0.206
	-	std_speed_yellow	−0.021	0.111	−0.187	0.147
	-	max_speed_yellow	0.070	0.149	−0.099	0.234
	-	min_speed_yellow	0.018	0.110	−0.150	0.185
dbq_errors	-	dbq_ordinaryViolations	0.577 ∗∗∗	$3.647 \times 10^{+10}$	0.447	0.676
	-	dbq_lapses	0.711 ∗∗∗	$1.084 \times 10^{+19}$	0.610	0.782
	-	mean_speed_yellow	0.163	0.624	−0.007	0.320
	-	std_speed_yellow	0.019	0.110	−0.149	0.185
	-	max_speed_yellow	0.251	7.678	0.084	0.399
	-	min_speed_yellow	0.011	0.109	−0.156	0.178
dbq_ordinaryViolations	-	dbq_lapses	0.337 ∗∗∗	286.333	0.175	0.474
	-	mean_speed_yellow	0.400 ∗∗∗	9693.128	0.244	0.529
	-	std_speed_yellow	−0.056	0.133	−0.221	0.113
	-	max_speed_yellow	0.545 ∗∗∗	$1.141 \times 10^{+9}$	0.409	0.650
	-	min_speed_yellow	0.188	1.134	0.019	0.342
dbq_lapses	-	mean_speed_yellow	0.038	0.119	−0.130	0.204
	-	std_speed_yellow	−0.050	0.127	−0.215	0.119
	-	max_speed_yellow	0.011	0.108	−0.157	0.177
	-	min_speed_yellow	8.082×10^{-4}	0.108	−0.166	0.168
mean_speed_yellow	-	std_speed_yellow	−0.684 ∗∗∗	$9.245 \times 10^{+16}$	−0.761	−0.577
	-	max_speed_yellow	0.706 ∗∗∗	$4.952 \times 10^{+18}$	0.605	0.779
	-	min_speed_yellow	0.809 ∗∗∗	$1.845 \times 10^{+29}$	0.737	0.858
std_speed_yellow	-	max_speed_yellow	−0.103	0.218	−0.265	0.066
	-	min_speed_yellow	−0.911 ∗∗∗	$2.642 \times 10^{+49}$	−0.935	−0.874
max_speed_yellow	-	min_speed_yellow	0.362 ∗∗∗	1081.461	0.203	0.496

* $BF_{10} > 10$, ** $BF_{10} > 30$, *** $BF_{10} > 100$

Table S8. Demographics - Bayesian Pearson Correlations

			Pearson's r	BF_{10}	Lower 95% CI	Upper 95% CI
exactAge	-	gender	−0.487 ∗∗∗	$5.519 \times 10^{+6}$	−0.602	−0.343
	-	mean_speed_yellow	−0.288 ∗∗	30.863	−0.432	−0.123
	-	std_speed_yellow	0.074	0.154	−0.096	0.237
	-	max_speed_yellow	−0.269∗	14.804	−0.415	−0.103
	-	min_speed_yellow	−0.104	0.220	−0.265	0.066
gender	-	mean_speed_yellow	−0.013	0.109	−0.180	0.154
	-	std_speed_yellow	0.159	0.582	−0.010	0.317
	-	max_speed_yellow	0.050	0.127	−0.119	0.215
	-	min_speed_yellow	−0.151	0.484	−0.308	0.019
mean_speed_yellow	-	std_speed_yellow	−0.684 ∗∗∗	$9.245 \times 10^{+16}$	−0.761	−0.577
	-	max_speed_yellow	0.706 ∗∗∗	$4.952 \times 10^{+18}$	0.605	0.779
	-	min_speed_yellow	0.809 ∗∗∗	$1.845 \times 10^{+29}$	0.737	0.858
std_speed_yellow	-	max_speed_yellow	−0.103	0.218	−0.265	0.066
	-	min_speed_yellow	−0.911 ∗∗∗	$2.642 \times 10^{+49}$	−0.935	−0.874
max_speed_yellow	-	min_speed_yellow	0.362 ∗∗∗	1081.461	0.203	0.496

* $BF_{10} > 10$, ** $BF_{10} > 30$, *** $BF_{10} > 100$
